# PascalX: a Python library for GWAS gene and pathway enrichment tests

**DOI:** 10.1093/bioinformatics/btad296

**Published:** 2023-05-03

**Authors:** Daniel Krefl, Alessandro Brandulas Cammarata, Sven Bergmann

**Affiliations:** Department of Computational Biology, University of Lausanne, Lausanne, Switzerland; Swiss Institute of Bioinformatics, Lausanne, Switzerland; Department of Computational Biology, University of Lausanne, Lausanne, Switzerland; Department of Computational Biology, University of Lausanne, Lausanne, Switzerland; Swiss Institute of Bioinformatics, Lausanne, Switzerland; Department of Integrative Biomedical Sciences, University of Cape Town, Cape Town, South Africa

## Abstract

**Summary:**

‘PascalX’ is a Python library providing fast and accurate tools for mapping SNP-wise GWAS summary statistics. Specifically, it allows for scoring genes and annotated gene sets for enrichment signals based on data from, both, single GWAS and pairs of GWAS. The gene scores take into account the correlation pattern between SNPs. They are based on the cumulative density function of a linear combination of $\chi^{2}$ distributed random variables, which can be calculated either approximately or exactly to high precision. Acceleration via multithreading and GPU is supported. The code of PascalX is fully open source and well suited as a base for method development in the GWAS enrichment test context.

**Availability and implementation:**

The source code is available at https://github.com/BergmannLab/PascalX and archived under doi://10.5281/zenodo.4429922. A user manual with usage examples is available at https://bergmannlab.github.io/PascalX/.

## 1 Introduction

Genome-wide association studies (GWASs) are well established for identifying links between genotypes and phenotypes. The results of such studies are usually freely shared in terms of summary statistics containing single SNP effect sizes. Aggregating such effects at the level of genes or pathways (i.e. annotated gene sets) map potential signals to biologically relevant entities, reducing the burden of multiple hypotheses testing and increasing power. There exist many aggregation methods, which differ by their statistical method, required input, approximation, and computational needs, c.f. de Leeuw et al. (2016) for a review.

Our original pathway scoring algorithm ‘Pascal’ (Lamparter et al. 2016) used sum of squared (normalized) effect sizes to score genes or pathways. In general, the cumulative distribution function (CDF) of such scores is a linear combination of $\chi^{2}$ variables under the null hypothesis, which can be computed efficiently. The linear combination coefficients are the eigenvalues of the SNP–SNP covariance matrix, whose off-diagonal elements are due to linkage disequilibrium, potentially inducing correlated individual SNP effects. The advantage of this method is that, like MAGMA (de Leeuw et al. 2015), its underlying statistic can be computed relatively fast and quasi-analytically, not requiring any approximations or perturbation-based estimates. Yet, in contrast to some other methods like ACAT (Liu et al. 2019), it does require an estimate of the SNP–SNP covariance matrix.

With the ever growing sample sizes used in current GWAS, also the signal strengths have been increasing. This can give rise to scores whose significance is so high that they cannot be computed accurately anymore with the double precision algorithms implemented in the original ‘Pascal’ software, resulting in unresolved rankings between the top genes.

The software described in this application note, called ‘PascalX’, solves this issue through a C++ multi-precision implementation of the CDF calculation (making use of the boost libraries, www.boost.org), interfaced via C Foreign Function Interface with a re-implementation of the original exact gene and pathway scoring of Lamparter et al. (2016) in Python. We also show that a saddle-point approximation (Kuonen 1999) is a viable alternative with an acceptable loss of accuracy (see online supplementary material), in particular if computation time is relevant. Our ‘PascalX’ implementation offers several further technical improvements over the original ‘Pascal’, namely intrinsic parallelization, possibility for GPU acceleration of linear algebra operations, and a new data storage model with fast random access. A detailed performance comparison to the original Pascal software can be found in the online supplementary material.

Furthermore, ‘PascalX’ also includes several new gene and pathway scoring methods. Specifically, it allow for cross-scoring using two sets of GWAS summary statistics using our recently proposed gene coherence and ratio enrichment tests (Krefl and Bergmann 2022). It also implements the possibility of not only selecting SNPs by window around gene transcription sites, but also for selecting SNPs by additional marker data. For instance, using the SNP to gene linking of Gazal et al. (2022), as demonstrated in Supplementary Figs S5–S7.

We provide ‘PascalX’ as fully open source python library, with a modern modular structure, extensive documentation, and a permissive AGPL-3.0 license. Our code components can easily be modified or replaced, making ‘PascalX’ an ideal code base for research on high-level analysis of GWAS summary statistics, including alternative scoring schemes, such as e.g. ACAT (Liu et al. 2019). We also provide a direct command line interface covering core functionalities, facilitating easy integration into production pipelines.

## 2 Features

### 2.1 CDF calculation

‘PascalX’ implements two common algorithms for the exact (up to a requested precision) calculation of the CDF of a linear combination of *N* random variables following a $\chi_{1}^{2}$ distribution, namely Ruben’s (Ruben 1962; Sheil and O’Muircheartaigh 1977; Farebrother 1984) and Davies’ algorithm (Davies 1973). While Ruben’s algorithm only supports positive coefficients, Davies’ algorithm also works for linear combinations with negative coefficients. For approximate calculation of the CDF, ‘PascalX’ offers the moment-based methods of Satterthwaite–Welch (Welch 1938; Satterthwaite 1946) and Imhof–Pearson (Pearson 1959; Imhof 1961), and calculation via saddle-point approximation (Kuonen 1999).

To the best of our knowledge, ‘PascalX’ is the first pathway scoring method using multi-precision arithmetic to achieve sufficient precision for application to modern GWAS data. Specifically, ‘PascalX’ can compute the CDF exactly up to a precision of 100 digits. We confirmed the correctness of the CDF calculations at high precision by cross-verifying Ruben’s and Davies’ algorithm against each other, see Supplementary Figs S1 and S2. Depending on the parameters of the linear combination, one of the algorithms may be strongly preferred over the other in run-time. In particular, multi-precision arithmetic is computationally expensive, and we do not know the needed precision for a particular CDF evaluation before-hand. Therefore, ‘PascalX’ utilizes a simple heuristic, switching automatically between algorithm and level of precision, to evaluate the CDF usually much faster than employing only one of the algorithms at fixed high precision (see Supplementary Section S1.4 for more details).

Both algorithms compute the CDF exactly up to a desired precision. This differs from a more commonly applied approximate calculation. So far the quality of such approximations has only been studied at low precision in Bodenham and Adams (2016), but there are no equivalent published results for the high-precision setting (i.e. beyond double precision). Using our high-precision implementation of the exact solution, we showed that the two moment-based approximations lead to substantial overestimation of significance in several parameter regimes, in particular for small *N*. In contrast, the saddle-point method yields a very good approximation, also for small *N* (see Supplementary Figs S3 and S4). Therefore, the saddle-point method is in general a good choice due to its superior performance and high accuracy (see Supplementary Figs S9–S12). ‘PascalX’ uses the saddle-point approximation as default.

### 2.2 Gene scoring

The gene scoring algorithm employed to test for GWAS gene enrichment has been originally introduced in Lamparter et al. (2016). ‘PascalX’ exclusively uses the sum of $\chi^{2}$ method, which is based on the test statistic for a gene *G* under the null assumption that $z∼N(0,\Sigma_{G})$. Here, $N_{G}$ denotes the number of SNPs in the gene region, $z_{i}$ the inverse normal transformed GWAS effect size *P*-value of the *i*th SNP and $\Sigma_{G}$ the SNP–SNP covariance matrix inferred from the genotype of a reference population. It can be shown that with $\lambda_{i}$ the *i*th eigenvalue of Σ. Hence, *T* follows a linear combination of $\chi_{1}^{2}$ distributions, and gene enrichment can therefore be tested for via the *P*-value $P=1-{\text{CDF}}_{\Xi }(T)$. The calculation of the CDF is described in Section 2.1. Benchmarking results for a set of standard GWAS and different choices of method to calculate the CDF are shown in Supplementary Fig. S13. Note that one can introduce an additional weighting of SNPs into the test statistic defined in Equation (1). The corresponding null distribution still follows a linear combination of $\chi_{1}^{2}$ distributions. Details are given in Supplementary Section S2.


(1) $$T_{G}=\sum_{i=1}^{N_{G}}z_{i}^{2},$$

$$T∼\sum_{i}\lambda_{i}[\chi_{i}^{2}]=:[\Xi ],$$

### 2.3 Cross-scoring

PascalX implements two novel cross GWAS gene coherence enrichment tests based on the product-normal distribution, described in detail in Krefl and Bergmann (2022). The first test, referred to as coherence test, is the statistics with *z* and *w* effect sizes of two different GWAS, both assumed to be following $N(0,\Sigma_{G})$ under the null. The second test, called ratio test, is given by or with *z* and *w* interchanged. As derived in Krefl and Bergmann (2022), the null distribution of these two new statistical tests can also be expressed in terms of a linear combination of $\chi_{1}^{2}$ distributions, albeit for these tests the linear combination contains also negative coefficients, such that only Davies’ algorithm can be applied for calculation of the corresponding CDF, see Supplementary Section S1.


$$X_{G}=\sum_{i=1}^{N_{G}}z_{i}w_{i},$$

$$R_{G}=\frac{\sum_{i=1}^{N_{G}}z_{i}w_{i}}{\sum_{i=1}^{N_{G}}z_{i}^{2}},$$

### 2.4 Pathway scoring

The pathway scoring algorithm PascalX makes use of to aggregate individual gene scores to pathway level scores is the gene fusion-based algorithm of Lamparter et al. (2016). In detail, genes in a pathway, which are in close proximity to each other, are fused to form a so-called meta-gene, and a corresponding gene score is calculated as for ordinary genes described in Section 2.2. This corrects for LD induced dependencies between the individual gene scores, such that one can test for pathway enrichment against a $\chi_{N}^{2}$ distribution under ranking and inverse transforming the individual gene *P*-values to $\chi_{1}^{2}$ distributed random variables. Since the main ingredient are the gene scores described in Section 2.2, the improvements in the original genescoring PascalX offers are directly inherited to the pathway scoring.

Since the pathway scoring is independent on the origin of the gene scores, pathway enrichment can also be tested for the cross-enrichment scores of Section 2.3.

### 2.5 Acceleration

#### 2.5.1 Multithreading

Computations on individual gene level with GWAS data are ideally suited for acceleration via parallelization, as computations for each gene can be performed independently from other genes. PascalX makes use of this and offers the option to parallelize computations via python multiprocessing. The performance gain achievable is illustrated in the Supplementary Figs S13–S15.

#### 2.5.2 GPU

The PascalX methodology requires the computation of large genotypic covariance matrices, and eigenvalue decompositions thereof. It is well known that such basic linear algebra matrix operations can be significantly speedup on specialized hardware, like graphic processor units. Therefore, PascalX supports GPU acceleration of these operations, making use of the ‘cupy’ library (Okuta et al. 2017). The performance gain is significant, as is illustrated in Supplementary Figs S13–S15.

#### 2.5.3 Storage

PascalX requires fast random access to SNP level genotypes of a reference population. Therefore, PascalX stores the genotypes in its own format, indexed by genomic position and variant id. Only indices are kept in memory and the genotypes are read on the fly from storage. In detail, the data for each individual SNP are stored serialized via pickle with additional zlib compression. The corresponding position on disk is stored in indices for genomic position and variant id for fast random access retrieval.

## Supplementary Material

btad296_Supplementary_Data

## Data Availability

As the data has been only used for the supplementary analysis in the supplement, the (rather long) data availability statement can be found in the Supplementary Material.
