# Supplementary material for: PascalX: a Python library for GWAS gene and pathway enrichment tests
Source: Bioinformatics. 2023 May 3;39(5):btad296. doi: 10.1093/bioinformatics/btad296 (PMC10185402; doi:10.1093/bioinformatics/btad296)
Supplement: btad296_Supplementary_Data [file btad296_supplementary_data.pdf]

PascalX - Supplementary information

Daniel Krefl[1,2,*], Alessandro B. Cammarata[1] and Sven Bergmann[1,2,3,*]

[1] *Department of Computational Biology, University of Lausanne, Switzerland*
[2] *Swiss Institute of Bioinformatics, Lausanne, Switzerland*
[3] *Dept. of Integrative Biomedical Sciences, University of Cape Town, South Africa*

February 2023

1 CDF calculation

1.1 Exact algorithms

Ruben's Ruben [1] showed that the cumulative distribution function (CDF) of a linear combination of χ^2 distributed random variables with positive coefficients can be expanded as an infinite series

$$\sum_{k=0}^{\infty} a_k \, F_{\chi^2_{m+2k}}(c/\beta)\,,$$

with $F_{\chi^2_{m+2k}}$ the CDF of a χ^2 distribution with $m+2k$ degrees of freedom and $\beta > 0$ an arbitrary constant. For $a_k \geq 0$ and $\sum_k a_k = 1$ the above sum constitutes a so-called (infinite) mixture representation. In particular, every $\beta \leq \lambda_{min}$ yields such a mixture representation, with λ_{min} denoting the smallest coefficient of the linear combination of χ^2 distributed random variables. Ruben showed that in the mixture case, the truncation error, and so the precision ϵ, can be bounded by

$$\left| 1 - \sum_{k=0}^{K-1} a_k \right| F_{\chi^2_{m+2K}}(c/\beta) \leq \epsilon\,,$$

with K the number of terms kept. Thereby, the coefficients a_k can be determined recursively, with the number of terms to be summed for a_k growing exponentially with k, but finite for given desired precision ϵ.

A first implementation of Ruben's algorithm can be found in [2] and an improved version by Farebrother in [3]. In particular, Farebrother improved numerical details and proposed to use a β which does not yield a mixture presentation, but has an improved rate of convergence. Namely,

$$\beta = \frac{2\lambda_{min}\lambda_{max}}{\lambda_{min} + \lambda_{max}}\,.$$

However, one should note that Farebrother still used the above mixture bound, and not the bound Ruben derived for general β. For both choices of β, the error bound at order k of the expansion

*Corresponding authors

contains a factor of $(\frac{\lambda_{max}}{\lambda_{min}} - 1)^k$. Hence, if $\lambda_{max} \gg \lambda_{min}$ the series converges very slowly.

Davies' Davies' algorithm [4, 5] is based on the numerical inversion of the characteristic function of a linear combination of χ^2 distributed random variables. It is more general than Ruben's algorithm, as it allows for negative coefficients and an additional normal distributed random variable. It also offers superior performance in specific regions of parameter space.

The number of integration terms for a given set of parameters can be estimated as follows. Since it will be sufficient for our purposes, we restrict the following discussion to the χ^2 case without the additional normal distributed term.

The relevant error bound of Davies for the summation reads in this case

$$\frac{c}{\prod_{i=1}^{N}(1 + 4(K + \frac{1}{2})^2\Delta^2\lambda_i^2)^{\frac{1}{4}}} < \frac{\epsilon}{2} \,,$$

with $c = \frac{10}{\pi}$, K the number of terms, Δ the integration interval and ϵ the desired accuracy. A weaker bound can be obtained by setting $\lambda_i = |\lambda|_{min}$. In this case we have

$$\frac{c}{(1 + 4(K + \frac{1}{2})^2\Delta^2|\lambda|_{min}^2)^{\frac{N}{4}}} < \frac{\epsilon}{2} \,,$$

and infer

$$\frac{1}{2\Delta|\lambda|_{min}}\sqrt{\frac{(2c)^{4/N}}{\epsilon^{4/N}} - 1} - \frac{1}{2} < K \,.$$

From this we can approximate that Davies' algorithm needs at most $K > \frac{(2c)^{2/N}\epsilon^{-2/N}}{2\Delta|\lambda|_{min}}$ integration terms. It remains to discuss Δ.

Davies fixes Δ as follows [4]

$$\Delta = \frac{2\pi}{|\psi'(u_*) - I|} \,,$$

with I the evaluation point of the CDF and ψ the logarithm of the moment generating function,

$$\psi(u) = -\frac{1}{2}\sum_{j=1}^{N}\log(1 + 2u\lambda_j) \,,$$

such that

$$\psi'(u) = \sum_{j=1}^{N}\frac{\lambda_j}{1 + 2u\lambda_j} \,.$$

The precise point u_* is not of importance for us, as we can estimate under the mild assumption $2u_*\lambda_{min} > -1$ via the logarithmic inequality $\frac{x}{1+x} \leq \log(1 + x) \leq x$ that

$$\psi'(u) \leq \sum_j \lambda_j \,.$$

Hence,

$$\Delta \geq \frac{2\pi}{|I - \sum_j \lambda_j|} \,,$$

and we arrive at the estimate of the number of integration terms K needed to obtain precision ϵ

$$\frac{|I - \sum_j \lambda_j|(2c)^{2/N}\epsilon^{-2/N}}{4\pi|\lambda|_{min}} \leq K\,. \tag{1}$$

We conclude that for sufficiently large N Davies' algorithm scales favorably due to the $\epsilon^{-2/N}$ dependence. However, for small N at high precision other algorithms may be preferred.

1.2 Approximations

A variety of approximations to the distribution Ξ of a linear combination of χ^2 distributed random variables exists, see for instance [6].

Satterthwaite-Welch One of the more well known methods is the Satterthwaite-Welch approximation [7, 8], which matches the first two moments to a Gamma distribution Γ. In detail [9],

$$\Xi \approx g\,\chi_h^2 \sim \Gamma(h/2, 2g)\,,$$

with

$$g := \frac{\sum_i d_i\lambda_i^2}{\sum_i d_i\lambda_i}\,, \quad h := \frac{\left(\sum_i d_i\lambda_i\right)^2}{\sum_i d_i\lambda_i^2}\,.$$

As before λ denote the coefficients of the linear combination and d_i denotes the ith degree-of-freedom parameter of the ith χ^2. In our application, all $d_i = 1$.

Imhof-Pearson's Following Pearson's work [10], Imhof presented the following $3rd$ order approximation to the cumulative density function of a linear combination of N χ_1^2 distributed random variables [11]:

$$\mathrm{cdf}_\Xi(x) \approx \mathrm{cdf}_{\chi_h^2}(y)\,,$$

with

$$h = \frac{c_2^3}{c_3^2}\,, \quad y = (x - c_1)\sqrt{h/c_2} + h\,, \quad c_j = \sum_{i=1}^N \lambda_i^j\,.$$

Note that Imhof presented a more general approximation formula, capturing the non-central and arbitrary degree of freedom case. For our purposes, however, the above simplified version is sufficient. In order not to confuse this approximation with Imhof's exact solution, we will refer to this third order approximation as Imhof-Pearson method, or often just as Pearson's method.

Saddle-point The saddle-point method uses the entire cumulant generating function and approximates the cumulative probability density function by, *c.f.,* [12],

$$\mathrm{cdf}_\Xi(x) \approx \frac{1}{2}\left(1 + \mathrm{erf}\left(\frac{w + \frac{1}{w}\log\left(\frac{v}{w}\right)}{\sqrt{2}}\right)\right)\,,$$

with erf the error function and

$$w = \mathrm{sgn}(\hat\zeta)\sqrt{2(\hat\zeta x - K(\hat\zeta)}\,, \quad v = \hat\zeta\sqrt{K''(\hat\zeta)}\,,$$

$$K(\zeta) = -\frac{1}{2}\sum_{i=1}^{N} \log\left(1 - 2\zeta\lambda_i\right) \ , \quad K'(\zeta) = \sum_{i=1}^{N} \frac{\lambda_i}{1 - 2\zeta\lambda_i} \ , \quad K''(\zeta) = 2\sum_{i=1}^{N} \frac{\lambda_i^2}{(1 - 2\zeta\lambda_i)^2} \ .$$

Note that it is required that $\zeta < \frac{1}{2}\min_i \lambda_i^{-1}$. The evaluation point $\hat{\zeta}$ is the saddle-point determined by the solution of $K'(\hat{\zeta}) = x$. In our implementation, we determine $\hat{\zeta}$ numerically via the Newton-Raphson method. The saddle-point method becomes numerically unstable for $\sum_i \lambda_i \approx x$ [12]. We therefore do not utilize the method if $\left|\frac{\sum_i \lambda_i - x}{x}\right| < 10^{-5}$.

Remark Note that the approximation solutions above are not suitable to approximate the cumulative distribution function of the product-normal distribution, which is the core ingredient of the cross-GWAS coherence test introduced in [13]. The reason being that in the case of the product-normal the sum over odd powers of λ_i vanishes, and therefore the above approximations are not well defined.

To our knowledge, the quality of the above approximations at very small p-values (below double precision) has not been evaluated in the literature, *c.f.*, [6]. We will fill this gap in the following section 1.3.

1.3 Implementation details and correctness verification

In order to have sufficient internal precision for our purposes, the implementation of Ruben's and Davies' algorithm in *PascalX* supports, besides standard *float64*, also *float128* and multi-precision arithmetic at 100 digits, making use of the *Boost* C++ libraries [14].

Since to our knowledge there is no other implementation which is able to evaluate the CDF of a linear combination of χ^2 random variables to such high precision, we verified the correctness of our implementation via cross-checking Ruben's and Davies' algorithm against each other.

In detail, we uniformly random sampled the number of linear combination terms from $[5, 500]$, coefficients from $[0.01, 1]$ and evaluation point from $[0.1, 1000]$. In total, we took $10,000$ samples. We evaluated for each sample one of the algorithms at set given requested precision, and verified with the other algorithm at higher requested and internal precision, up to requested accuracy of 10^{-32}. The results are shown in figure 1, confirming the correctness and accuracy of the CDF calculation.

In order to verify the correctness at higher levels of precision, we in addition cross verified the two algorithms against each other at 100 digits internal precision. The results are illustrated in figure 2, confirming accuracy up to $\sim 2 \times 10^{-96}$.

Finally, we also compared the exact calculation against the above discussed approximate solutions, see figures 3 and 4. We observe that the approximation solutions of Satterthwaite-Welch and Imhof-Pearson systematically over estimate $-\log_{10}$ transformed p-values, with magnitude increasing with decreasing number of linear combination terms in the CDF. In contrast, the saddle-point method yields an excellent approximation, also at small N and for very small p-values.

1.4 Automatic algorithm selection

PascalX invokes a simple heuristic to select between Ruben's and Davies' algorithm, which we will refer to as *auto* mode. The heuristic is as follows.

Since the required precision is not known up front, the CDF is first approximated via Imhof-Pearson's method. The resulting p-value determines the initial requested precision. The number of needed integration terms for Davies' algorithm is estimated via relation (1), and the ratio $\lambda_{max}/\lambda_{min}$ is determined. If the ratio is larger than a preset number, and there are more than a specified number of terms in the CDF, and the number of integration terms is smaller than a preset amount at a specific precision level, then Davies' algorithm is used, otherwise Ruben's. If the evaluation fails the required precision or number of integration terms is increased and the evaluation is repeated for a given number of tries.

2 Gene and Pathway scoring

2.1 Trait data

Our analysis of the performance of PascalX is based on eight common GWAS traits, listed in table 1. For links to the utilized public GWAS summary statistics and the used trait abbreviations we refer to the *"Data availability"* section further below. Note that three of the considered GWAS (Inflammatory bowel disease, Rheumatoid arthritis and Alzheimer) have $\sim 10M$ SNPs, while the other GWAS are considerably smaller with $\sim 2M$ SNPs.

2.2 PascalX settings

Per default settings, PascalX considers all SNPs in a window extending 50kb from the gene transcription start and end positions. Minor allele frequency threshold for inclusion of a SNP is taken to be 0.05 and eigenvalue cutoff is set to keep 99% of total gene variance. We considered all protein coding genes taken from Ensembl Biomart (https://www.ensembl.org/).

2.3 Weighted Pascal

The test statistic given in equation (1) of the main text can be generalized by introducing additional weights w_i to the SNP contributions, *i.e.*,

$$T_G^w = \sum_{i=1}^{N_G} w_i\, z_i^2 \,,$$

with N_G denoting the number of SNPs in the region corresponding to the gene G. We require that all $w_i > 0$. Recall from the main text that we assume that $z \sim \mathcal{N}(0, \Sigma_G)$ with Σ_G the SNP-SNP covariance matrix inferred from a reference population. Introducing a diagonal weight matrix W, we can write equivalently,

$$T_G^w = z^t W z = (W^{1/2} z^t)\,(W^{1/2} z) := \hat{z}^t \hat{z} \,.$$

From the affine transform property of the multivariate normal distribution we infer that

$$\hat{z} \sim \mathcal{N}\left(0, (W^{1/2})\,\Sigma_G\,(W^{1/2})\right).$$

Similar as in the original derivation of the unweighted case, *c.f.* [15], we can conclude that

$$T_G^w \sim \sum_i \hat{\lambda}_i \, [\chi_1^2] \,, \tag{2}$$

with $\hat{\lambda}_i$ the eigenvalues of $\widehat{\Sigma}_G := (W^{1/2}) \, \Sigma_G \, (W^{1/2})$.

2.4 Regularisation

The SNP-SNP covariance matrix Σ_G can be poorly conditioned. This is of particular concern if a gene region contains more SNPs than samples are available to calculate Σ_G. However, an advantage of the sum of χ_1^2 based statistic utilized in this work is that regularization can easily be achieved by introducing an eigenvalue cutoff in the weighted χ_1^2 expansion (2). In our implementation, we drop vanishing or negative eigenvalues and keep only eigenvalues up to a user specified total variance (99% by default). We tested the impact of varying reference panel size and observe that the impact is relatively minor for significant p-values compared to the impact of approximating the cumulative distribution function with the best moment based method, see figure 8.

2.5 Exact vs. approximate solutions

We tested for a panel of standard GWAS, listed in table 1, how well the approximate CDF calculation performs for gene scoring. We used the PascalX default settings stated in the previous subsection 2.2 and considered only protein coding genes. We observe that the moment based methods of Satterthwaite-Welch and Imhof-Pearson in general overestimate the $-\log_{10}$ transformed gene p-values, as visible from the plots in figure 9. In contrast, the saddle-point approximation yields p-values well calibrated to the exact calculation. In order to see if also the relative order of the genes is impacted, we calculated the Spearmann correlation between the exactly and approximately calculated gene p-values for different p-value ranges. The results are listed in table 11. The ranking is not fully preserved under approximation via the moment based methods for p-values below 10^{-16}. In contrast, the saddle-point method preserves very well the ordering of genes, also for small p-values.

We also verified that under pathway aggregation the saddle-point approximation is best calibrated to the exact computation, see figure 11 (we tested against all pathways of MSigDB v7.4). In addition, we confirmed via calculating Spearman correlations that the ranking of pathways for smaller p-values is impacted by the moment based approximation methods, while for the saddle-point method the impact is minor, *c.f.*, table 12.

2.6 Run-time evaluation

For the benchmarking of computation time the PascalX settings detailed in section 2.2 have been used. All experiments have been conducted on a server equipped with two Intel Xeon Gold 5220 CPUs (2.2 GHz, 18 cores each), 512 GB RAM, NVIDIA RTX A5000 GPU (24 GB RAM), a RAID 0 of four Samsung 970 EVO Plus NVME drives for storage of reference panel and GWAS data, and Ubuntu 18.04.6 LTS as operating system. Since we cannot rule out that other users might have used the server during the benchmarking, the results below should be taken as an indicative upper bounds rather than as absolute estimates.

2.6.1 Generalities

The gene scoring computation can be split into three distinct parts. First, loading of genotype reference data for a gene window from disk. Second, linear algebra operations to calculate the gene window SNP covariance matrix and corresponding eigenvalue decomposition. Third, calculation of the corresponding weighted χ^2 CDF. Note that the reference panel size N only enters via disk IO with scaling $\mathcal{O}(N * S)$ and in the covariance matrix calculation, scaling $\mathcal{O}(N * S^2)$, with S the number of SNPs in the gene window. The runtime of the third part only depends on S and scales $\mathcal{O}(S^3)$. Hence, linear algebra operation runtime is mainly determined by the number of SNPs in the gene window under consideration. Therefore, we expect that we profit most of GPU acceleration for highly imputed GWAS. The runtimes and throughputs in genes per second stated in this work always refer to the complete computation, covering all three parts sketched above.

Note that the system level BLAS/LAPACK libraries invoked on our testing server are multi-threaded and make already use by itself of the high core count of the server. The presented CPU benchmarks for varying parallel setting are therefore biased.

Memory consumption of PascalX is determined by the size of the GWAS summary statistics considered and the size of reference panel used (number of SNPs included). PascalX keeps a SNP to disk position index in memory per active chromosome. We therefore recommend to have at least 4GB of memory for each CPU core in use.

2.6.2 Gene scoring

Run-times of PascalX for gene scoring using exact and approximate CDF calculation for various levels of parallelization and GPU utilization are given in tables 2,3, 4 and 5 in genes per second, and illustrated for absolute runtimes in figure 13.

For each algorithm and setting tested we plot the run-times as bar plot generated from the individual run-times of the 8 GWAS listed in table 1. We observe that the exact calculation possesses significantly longer and more varied run-times compared to the approximation methods. This is likely due to the fact that for a small subset of genes both Ruben's and Davies' algorithm struggle to converge as they are not well suited for the corresponding CDF calculation. We observe that the mean run-time decreases with increasing parallelization and that there is a significant improvement in mean run-time with GPU utilization. The best results are obtained for the approximation methods with GPU acceleration and parallel setting of eight. Due to GPU memory being a limiting factor, we did not try a higher parallel setting with a single GPU.

2.6.3 X scoring

Results for the benchmarking of the cross coherence scorer of PascalX are listed in table 6 and illustrated in figure 14. Cross scores can only be calculated exactly via Davies' method. We tested each setting at hand of all unique pairs of GWAS given in table 1. As for the gene scoring above, we observe that GPU acceleration of linear algebra operations gives a noticeable performance boost.

2.6.4 Pathway scoring

Benchmarking results for pathway scoring (with gene fusion) are given in tables 7 and 8, and are summarized in figure 15.

Trait	# Subjects	# SNPs	Reference
Alzheimer	63,926	9,046,216	Kunkle, B. et al. (2019) [20]
Body mass index	249,796	2,407,537	Speliotes, E. et al. (2010) [21]
Coronary artery disease	194,427	2,420,294	CARDIoGRAMplusC4D (2013) [22]
Height	183,727	2,469,114	Lango, A. et al. (2010) [23]
Inflammatory bowel disease	86,640	11,553,259	IIBDGC [24, 25]
Rheumatoid arthritis	25,500	2,554,113	Stahl, E. et al. (2010) [26]
Schizophrenia	150,064	10,694,907	PGC (2014) [27]
Type-II diabetes	69,033	2,473,390	Morris, A. et al. (2012) [28]

Table 1: List of GWAS used with population size, number of SNPs and reference to original study.

Gene scoring: **Auto (exact)**								
Trait	$p{=}1$	$p{=}2$	$p{=}4$	$p{=}22$	$p{=}1$ gpu	$p{=}2$ gpu	$p{=}4$ gpu	$p{=}8$ gpu
ALZ	1.9	2.1	2.0	2.3	2.2	2.8	2.9	3.1
BMI	7.2	10.6	16.1	18.2	11.4	23.3	39.5	58.2
CAD	8.3	12.3	16.0	20.6	11.1	23.5	42.7	69.4
H	6.5	10.3	14.9	16.8	11.1	20.7	39.7	58.9
IBD	2.0	2.1	2.2	2.5	3.9	5.2	6.4	6.8
RA	2.2	2.4	2.4	2.7	2.5	2.8	3.0	3.1
SCZ	4.1	5.3	5.6	6.0	8.8	16.7	27.2	41.2
T2D	8.3	12.6	16.0	18.7	12.7	23.3	41.7	68.3
**median**	5.3	7.8	10.3	11.4	10.0	18.7	33.4	49.7
**mean**	5.1	7.2	9.4	11.0	8.0	14.8	25.4	38.6

Table 2: Throughput in genes per second for PascalX gene scoring with the auto (exact) method. $^{p=n}$ denotes the number of cores to use set for PascalX and $_{gpu}$ denotes usage of GPU for acceleration of linear algebra operations. Trait codes are defined in the section *"Data availability"*.

Gene scoring: **Satterthwaite**								
Trait	$p{=}1$	$p{=}2$	$p{=}4$	$p{=}22$	$p{=}1$ gpu	$p{=}2$ gpu	$p{=}4$ gpu	$p{=}8$ gpu
ALZ	4.3	5.9	6.5	7.6	7.2	11.1	18.9	28.3
BMI	8.4	13.1	18.8	23.8	10.8	16.4	30.4	54.1
CAD	8.5	13.3	18.7	24.1	11.1	16.9	30.6	54.9
H	8.2	13.3	8.4	18.6	10.8	12.2	30.4	54.5
IBD	4.4	5.8	6.6	7.4	7.3	11.2	19.1	28.6
RA	8.0	12.9	18.1	23.0	10.7	16.5	30.1	53.0
SCZ	3.7	5.9	6.7	7.8	7.3	11.0	18.9	28.6
T2D	8.5	13.3	20.0	23.4	11.1	16.9	18.1	54.4
**median**	8.1	13.0	13.2	20.8	10.7	14.3	24.6	53.5
**mean**	6.7	10.4	13.0	17.0	9.5	14.0	24.6	44.6

Table 3: Description as for table 2, but with Satterthwaite method.

Gene scoring: **Pearson**								
Trait	p=1	p=2	p=4	p=22	p=1 gpu	p=2 gpu	p=4 gpu	p=8 gpu
ALZ	4.0	5.8	7.2	10.1	6.5	11.4	18.6	28.2
BMI	7.8	12.6	18.7	31.3	9.3	17.3	29.8	53.0
CAD	7.9	12.7	18.8	31.5	9.5	17.8	30.6	53.7
H	7.5	12.5	18.8	30.9	9.7	16.9	29.4	52.2
IBD	4.1	5.8	7.2	9.8	6.1	11.9	18.7	27.7
RA	7.7	12.0	18.2	29.4	9.5	17.3	29.0	52.0
SCZ	3.9	5.6	7.2	10.1	6.1	11.0	17.5	27.8
T2D	7.8	12.7	19.3	32.1	9.5	17.7	31.4	53.3
**median**	7.6	12.3	18.5	30.2	9.4	17.1	29.2	52.1
**mean**	6.3	10.0	14.4	23.1	8.3	15.2	25.6	43.5

Table 4: Description as for table 2, but with Pearson method.

Gene scoring: **Saddle**								
Trait	p=1	p=2	p=4	p=22	p=1 gpu	p=2 gpu	p=4 gpu	p=8 gpu
ALZ	4.3	5.7	6.4	7.5	6.7	11.8	19.9	29.5
BMI	8.1	12.6	18.3	23.1	10.0	18.0	31.9	57.1
CAD	8.2	12.9	18.6	23.9	10.1	18.0	32.9	58.7
H	8.0	12.2	18.3	23.0	10.0	17.9	32.3	57.8
IBD	4.3	5.7	6.5	7.7	6.8	11.8	20.0	29.3
RA	8.0	12.5	17.9	22.6	10.0	17.7	31.9	56.5
SCZ	4.3	5.8	6.5	7.6	6.7	11.7	19.4	29.7
T2D	8.1	13.0	18.6	24.1	10.2	18.1	32.7	58.2
**median**	8.0	12.4	18.1	22.8	10.0	17.8	31.9	56.8
**mean**	6.6	10.0	13.9	17.5	8.8	15.6	27.6	47.1

Table 5: Description as for table 2, but with Saddle method.

Cross scoring: **Davies**					
Trait	$^{p=4}$	$^{p=8}$	$^{p=22}$	$^{p=4}_{gpu}$	$^{p=8}_{gpu}$
ALZ – CAD	23.1	34.9	35.8	32.3	64.3
ALZ – RA	26.8	38.6	42.2	34.0	65.1
ALZ – SCZ	11.3	13.8	12.8	19.2	35.1
ALZ – IBD	9.3	10.1	10.8	15.4	29.6
ALZ – T2D	31.0	52.5	63.0	36.3	76.9
CAD – RA	26.9	40.6	44.6	33.5	63.5
CAD – SCZ	25.5	36.3	40.0	31.3	58.
CAD – IBD	23.5	36.6	38.8	31.0	68.4
CAD – T2D	31.3	53.6	66.8	36.8	78.5
RA – SCZ	27.8	41.6	49.2	32.6	63.2
RA – IBD	24.8	39.2	49.1	31.3	59.9
RA – T2D	25.4	42.7	41.7	33.5	65.7
SCZ – IBD	10.6	14.0	13.4	17.9	32.3
SCZ – T2D	30.4	47.6	68.2	35.3	63.2
IBD – T2D	30.9	46.1	60.3	35.5	64.2
**median**	25.5	39.2	42.2	32.6	63.5
**mean**	23.9	36.5	42.5	30.4	59.2

Table 6: Throughput in genes per second for cross scoring based on Davies method. $^{p=n}$ denotes the number of cores to use set for PascalX and $_{gpu}$ denotes usage of GPU for acceleration of linear algebra operations. Trait codes are defined in the section *"Data availability"*.

Pathway scoring: **Auto (exact)**			
Trait	$^{p=22}$	$^{p=4}_{gpu}$	$^{p=8}_{gpu}$
ALZ	0.5	0.3	0.3
BMI	8.3	10.8	14.9
CAD	6.4	12.3	16.6
H	5.8	5.6	6.8
IBD	0.4	0.2	0.3
RA	0.1	0.1	0.1
SCZ	2.0	5.7	7.7
T2D	7.7	8.7	17.6
**median**	3.9	5.7	7.3
**mean**	3.9	5.5	8.1

Pathway scoring: **Satterthwaite**			
Trait	$^{p=22}$	$^{p=4}_{gpu}$	$^{p=8}_{gpu}$
ALZ	2.6	4.1	6.6
BMI	10.6	12.1	17.5
CAD	10.4	12.0	17.1
H	10.4	11.9	17.8
IBD	3.1	7.3	9.5
RA	9.2	12.1	18.0
SCZ	2.2	5.7	7.1
T2D	11.1	8.2	17.7
**median**	9.8	10.0	17.3
**mean**	7.4	9.2	13.9

Table 7: Throughput in pathways per second for pathway scoring with the auto (exact) method (left table) and Satterthwaite method (right table). $^{p=n}$ denotes the number of cores to use set for PascalX and $_{gpu}$ denotes usage of GPU for acceleration of linear algebra operations. Trait codes are defined in the section *"Data availability"*.

Pathway scoring: **Pearson**			
Trait	p=22	p=4 gpu	p=8 gpu
ALZ	3.8	7.2	8.8
BMI	11.5	13.0	18.5
CAD	11.7	12.9	19.2
H	11.5	12.9	17.4
IBD	4.3	7.9	10.1
RA	11.2	12.8	18.9
SCZ	3.5	7.0	8.8
T2D	11.5	12.1	19.2
**median**	11.4	12.5	18.0
**mean**	8.6	10.7	15.1

Pathway scoring: **Saddle**			
Trait	p=22	p=4 gpu	p=8 gpu
ALZ	3.3	6.9	9.1
BMI	11.5	12.9	19.0
CAD	11.2	12.9	18.9
H	11.6	12.3	18.2
IBD	4.3	7.8	10.2
RA	10.9	12.5	18.8
SCZ	3.5	6.8	8.9
T2D	11.8	13.0	19.2
**median**	11.1	12.4	18.5
**mean**	8.5	10.6	15.3

Table 8: Description as in table 7, but with Pearson method (left table) and Saddle method (right table).

For the approximation methods we observe that GPU usage yields a significant performance boost. Interestingly, we do not observe such a boost for the exact calculation. Furthermore, the exact calculation takes significantly longer to complete than the approximation methods. A likely explanation is that in the pathway calculation we have far more (meta)-genes for which the exact CDF calculation takes very long to converge, outweighing the GPU speedup of the linear algebra operations.

2.7 Benchmark against previous implementation

We compare our new PascalX implementation against the original weighted χ^2 Pascal implementation of [15]. Note that we used for this analysis the reference panel and gene annotation delivered with the original Pascal also for PascalX. We used the default original Pascal setting, but set the original Pascal to use all SNPs in the genome region, as is the case for PascalX. As gene scoring method we used the saddle point approximation for PascalX. The results for weighted χ^2 based genescoring are shown in figure 16. We clearly observe that the original Pascal starts to break down around a $-\log_{10}$ p-value of 12. Below that double precision induced threshold, we observe a very good concordance between Pascal and PascalX calculated p-values. A runtime comparison can be found in table 9. We observe that in the single core setting PascalX is more than twice as fast as the original Pascal implementation. With GPU acceleration we are almost three times as fast. Increasing the core count, we can easily achieve more than a factor of ten. Note that the results presented in table 9 are not directly comparable to the benchmarking presented for just PascalX, as the latter is based on a larger (sample wise) and more extensive (SNP wise) reference panel.

Pathway scores are plotted against each other in figure 17. Corresponding qq-plots can be found in figure 18. We observe that the new PascalX implementation appears to yield less inflated results than the original Pascal implementation. Furthermore, we observe at hand of the highly powered GWAS for Alzheimer and Rheumatoid arthritis that PascalX's ability to resolve gene scores at higher precision impacts the pathway scores. Note that the importance of being able to compute highly significant genes is also visible in figure 11. In this figure we observe as well for Rheumatoid arthritis that the pathways using gene scores computed via the saddle point approximation seem to deviate more than usual from the pathways computed with the exact gene scores. The reason being that

Trait	Pascal	PascalX$^{p=1}$	PascalX$^{p=1}_{gpu}$	PascalX$^{p=8}$	PascalX$^{p=8}_{gpu}$
Alzheimer	3.0	10.4	17.0	13.8	56.5
Body mass index	15.3	26.7	33.6	48.1	161.3
Coronary artery disease	13.4	25.8	33.4	49.0	155.3
Height	10.6	26.5	33.5	47.8	165.2
Inflammatory bowel disease	2.7	9.8	16.8	13.4	55.6
Rheumatoid arthritis	11.3	25.2	32.9	45.8	159.4
Schizophrenia	4.1	11.3	18.3	14.9	61.8
Type-II diabetes	13.4	26.6	33.5	49.8	163.7
**median**	11.0	25.5	33.2	46.8	157.4
**mean**	9.4	20.3	27.4	35.3	122.4
**Δ mean (%)**		**+116**	**+192**	**+276**	**+1202**

Table 9: Benchmark of weighted χ^2 based genescoring of original Pascal implementation against PascalX. Results are given in genes per second. The Δ mean row gives the difference to the original Pascal in %. $^{p=n}$ denotes the number of cores to use set for PascalX and $_{gpu}$ denotes usage of GPU for acceleration of linear algebra operations. Note that for this analysis the reference panel of the original Pascal has been used (1K Genome Project phase 1, GRCh37).

this GWAS has a significant number of genes above the exact computation threshold of 100 digits. As the saddle point methods is able to resolve the gene ordering above 100 digits, we expect that pathway scores are impacted. Note also, that as an immune system related trait, it is expected that significant genes of RA are more commonly found in pathways.

3 List of tables

1. List of GWAS used.
2. Gene scoring throughput for Auto method.
3. Gene scoring throughput for Satterthwaite method.
4. Gene scoring throughput for Pearson method.
5. Gene scoring throughput for Saddle method.
6. Cross scoring throughput.
7. Pathway scoring throughput for Auto and Satterthwaite methods.
8. Pathway scoring throughput for Pearson and Saddle methods.
9. Gene scoring benchmark of original Pascal implementation against PascalX.
10. Pathway scoring benchmark of original Pascal implementation against PascalX.
11. Spearman correlations between exact and approximate calculation of gene scores.
12. Spearman between exact and approximate calculation of pathway scores.

Trait	Pascal	PascalX$^{p=1}$	PascalX$^{p=1}_{gpu}$	PascalX$^{p=8}$	PascalX$^{p=8}_{gpu}$
Alzheimer	0.1	0.3	0.8	0.5	1.8
Body mass index	0.5	1.5	1.4	2.2	9.1
Coronary artery disease	0.5	1.4	1.7	2.4	9.2
Height	0.4	1.5	2.0	2.0	9.3
Inflammatory bowel disease	0.1	0.3	0.8	0.4	1.7
Rheumatoid arthritis	0.5	1.4	1.9	2.6	8.4
Schizophrenia	0.1	0.4	0.9	0.6	2.2
Type-II diabetes	0.6	1.5	2.0	2.7	9.3
**median**	0.5	1.4	1.6	2.1	8.8
**mean**	0.4	1.0	1.4	1.7	6.4
**Δ mean (%)**		**+150**	**+250**	**+324**	**+1500**

Table 10: Benchmark of weighted χ^2 based pathway scoring of original Pascal implementation against PascalX. Results are given in pathways per second. The Δ mean row gives the difference to the original Pascal in %. $^{p=n}$ denotes the number of cores to use set for PascalX and $_{gpu}$ denotes usage of GPU for acceleration of linear algebra operations. Note that for this analysis the reference panel of the original Pascal has been used (1K Genome Project phase 1, GRCh37).

4 List of figures

1. Cross verification of Davies' and Ruben's algorithm with control.
2. Cross verification of Davies' and Ruben's algorithm at 100 digits precision.
3. Approximative methods against exact solution under varying number of linear combination terms.
4. Approximative methods against exact solution.
5. Gene scores for fixed window versus cS2G SNP to gene linking.
6. Pathway scores for fixed window versus cS2G SNP to gene linking.
7. QQ-plots for pathway scores based on fixed window and based on cS2G SNP to gene linking.
8. Gene score differences under varying reference panel and method.
9. Exact against approximate gene score calculation.
10. QQ-plots for different gene scoring methods.
11. Exact against approximate pathway score calculation.
12. QQ-plots for different pathway scoring methods.
13. Gene scoring performance benchmark.
14. X scoring performance benchmark.
15. Pathway scoring performance benchmark.
16. Gene scores for original Pascal implementation against PascalX.

17. Pathway scores for original Pascal implementation against PascalX.

18. QQ-plots for original Pascal and PascalX based pathway scores.

Code availability

We used PascalX v0.0.4 available from Zenodo [16] and on GitHub under the url https://github.com/BergmannLab/PascalX. For the comparison with the original Pascal implementation of [15] we used the code available at https://www2.unil.ch/cbg/index.php?title=Pascal.

Data availability

Gene annotation We considered only protein coding genes. Gene annotation can be downloaded from Ensemble BioMart https://www.ensembl.org/ (we used release 104).

Reference panel For this work we used the European subpopulation of the 1K Genome Project [17] as reference panel to estimate the SNP-SNP correlations. The data can be obtained from https://www.internationalgenome.org/. We mainly used the 632 european samples of the 30x high coverage GRCh38 release of [18], but for the comparison to the original Pascal implementation we used the GRCh37 phase 1 release with 379 european samples.

Pathway set We used the MSigDB v7.4 database to perform pathway enrichment tests. The data can be downloaded at https://www.gsea-msigdb.org/gsea/msigdb/.

cS2G The SNP to gene linking data of the cS2G method of [19] can be downloaded at https://alkesgroup.broadinstitute.org/cS2G/cS2G_1000GEUR/.

GWAS The GWAS summary statistics used in this work have been published by the authors of the cited original studies, and have been retrieved from the following websites:

Alzheimer (ALZ): [20]
https://www.ebi.ac.uk/gwas/studies/GCST007511

Body mass index (BMI): [21]
https://portals.broadinstitute.org/collaboration/giant/index.php/GIANT_consortium_data_files

Coronary artery disease (CAD): [22]
http://www.cardiogramplusc4d.org/data-downloads/

Height (H): [23]
https://www.ebi.ac.uk/gwas/publications/20881960

Inflammatory bowel disease (IBD): [24, 25]
https://www.ibdgenetics.org/downloads.html

Rheumatoid arthritis (RA): [26]
https://www.broadinstitute.org/ftp/pub/rheumatoid_ arthritis/Stahl_ etal_ 2010NG/

Schizophrenia (SCZ): [27]
https://www.med.unc.edu/pgc/download-results/

Type-II diabetes (T2D): [28]
http://www.diagram-consortium.org/downloads.html

References

[1] H. Ruben, "Probability Content of Regions Under Spherical Normal Distributions, IV: The Distribution of Homogeneous and Non-Homogeneous Quadratic Functions of Normal Variables." Ann. Math. Statist. Volume 33, Number 2 (1962), 542-570.

[2] J. Sheil and I. O'Muircheartaigh, "Algorithm AS 106: The Distribution of Non-Negative Quadratic Forms in Normal Variables Journal of the Royal Statistical Society." Series C (Applied Statistics) Vol. 26, No. 1 (1977), pp. 92-98

[3] "Algorithm AS 204: The Distribution of a Positive Linear Combination of χ^2 Random Variables." Journal of the Royal Statistical Society. Series C (Applied Statistics) Vol. 33, No. 3 (1984), pp. 332-339

[4] R. B. Davies, "Numerical Inversion of a Characteristic Function." Biometrika, Vol. 60, No. 2 (Aug., 1973), pp. 415-417

[5] Davies R. B. "Algorithm AS 155: The Distribution of a Linear Combination of χ^2 Random Variables." Journal of the Royal Statistical Society. Series C (Applied Statistics) Vol. 29, No. 3 (1980), pp. 323-333 doi:10.2307/2346911

[6] Dean A. Bodenham and Niall M. Adams, "A comparison of efficient approximations for a weighted sum of chi-squared random variables." Statistics and Computing volume 26, pages917–928(2016)

[7] B. L. Welch, "THE SIGNIFICANCE OF THE DIFFERENCE BETWEEN TWO MEANS WHEN THE POPULATION VARIANCES ARE UNEQUAL." Biometrika, Volume 29, Issue 3-4, February 1938, Pages 350–362, doi.org/10.1093/biomet/29.3-4.350

[8] F. E. Satterthwaite, "An Approximate Distribution of Estimates of Variance Components." Biometrics Bulletin, Vol. 2, No. 6 (Dec., 1946), pp. 110-114, International Biometric Society doi.org/10.2307/3002019

[9] G. E. P. Box, "Some Theorems on Quadratic Forms Applied in the Study of Analysis of Variance Problems, I. Effect of Inequality of Variance in the One-Way Classification." Ann. Math. Statist., Volume 25, Number 2 (1954), 290-302.

[10] Pearson, E. S., "Note on an Approximation to the Distribution of Non-Central χ^2," Biometrika, Dec., 1959, Vol. 46, No. 3/4 (Dec., 1959), p. 364, Oxford University

[11] Imhof, J. P., "Computing the Distribution of Quadratic Forms in Normal Variables," Biometrika, Dec., 1961, Vol. 48, No. 3/4 (Dec., 1961), pp. 419-426, Oxford University Press

[12] Kuonen, D., "Saddlepoint approximations for distributions of quadratic forms in normal variables." Biometrika, 1999, 86, 4, pp. 929-935

[13] Krefl D, Bergmann S (2022), "Cross-GWAS coherence test at the gene and pathway level." PLOS Computational Biology 18(9): e1010517. doi.org/10.1371/journal.pcbi.1010517

[14] Boost organization, Boost C++ libraries, https://www.boost.org/

[15] Lamparter D., Marbach D., Rueedi R., Kutalik Z., Bergmann S., "Fast and Rigorous Computation of Gene and Pathway Scores from SNP-Based Summary Statistics." *PLOS Computational Biology*, doi.org/10.1371/journal.pcbi.1004714

[16] Krefl, D. and Bergmann, S., "PascalX". (2021). Zenodo. doi.org/10.5281/zenodo.4429921

[17] The 1000 Genomes Project Consortium "A global reference for human genetic variation," Nature 526, 68-74 (01 October 2015) doi.org/10.1038/nature15393

[18] Marta Byrska-Bishop et. al., "High coverage whole genome sequencing of the expanded 1000 Genomes Project cohort including 602 trios." doi.org/10.1101/2021.02.06.430068

[19] Gazal, S., Weissbrod, O., Hormozdiari, F. et al. "Combining SNP-to-gene linking strategies to identify disease genes and assess disease omnigenicity." Nat Genet 54, 827–836 (2022). doi.org/10.1038/s41588-022-01087-y

[20] Kunkle, Brian W., Benjamin Grenier-Boley, Rebecca Sims, Joshua C. Bis, Vincent Damotte, Adam C. Naj, Anne Boland, et al, "Genetic Meta-Analysis of Diagnosed Alzheimer's Disease Identifies New Risk Loci and Implicates Aβ, Tau, Immunity and Lipid Processing." Nature Genetics 51, no 3 (March 2019): 414 30 doi.org/10.1038/s41588-019-0358-2

[21] Speliotes, Elizabeth K., Cristen J. Willer, Sonja I. Berndt, Keri L. Monda, Gudmar Thorleifsson, Anne U. Jackson, Hana Lango Allen, et al, "Association Analyses of 249,796 Individuals Reveal 18 New Loci Associated with Body Mass Index." Nature Genetics 42, no 11 (November 2010): 937 48 doi.org/10.1038/ng.686

[22] The CARDIoGRAMplusC4D Consortium, "Large-scale association analysis identifies new risk loci for coronary artery." Nature genetics 45, no 1 (January 2013): 25 33 doi.org/10.1038/ng.2480

[23] Lango Allen, Hana, Karol Estrada, Guillaume Lettre, Sonja I. Berndt, Michael N. Weedon, Fernando Rivadeneira, Cristen J. Willer, et al, "Hundreds of Variants Clustered in Genomic Loci and Biological Pathways Affect Human Height." Nature 467, no 7317 (October 2010): 832 38 doi.org/10.1038/nature09410

[24] Goyette, Philippe, Gabrielle Boucher, Dermot Mallon, Eva Ellinghaus, Luke Jostins, Hailiang Huang, Stephan Ripke, et al, "High-Density Mapping of the MHC Identifies a Shared Role for HLA-DRB1*01:03 in Inflammatory Bowel Diseases and Heterozygous Advantage in Ulcerative Colitis." Nature Genetics 47, no 2 (February 2015): 172 79 doi.org/10.1038/ng.3176

[25] Liu, Jimmy Z., Suzanne van Sommeren, Hailiang Huang, Siew C. Ng, Rudi Alberts, Atsushi Takahashi, Stephan Ripke, et al, "Association Analyses Identify 38 Susceptibility Loci for Inflammatory Bowel Disease and Highlight Shared Genetic Risk across Populations." Nature Genetics 47, no 9 (September 2015): 979 86 doi.org/10.1038/ng.3359

[26] Stahl, Eli A., Soumya Raychaudhuri, Elaine F. Remmers, Gang Xie, Stephen Eyre, Brian P. Thomson, Yonghong Li, et al, "Genome-Wide Association Study Meta-Analysis Identifies Seven New Rheumatoid Arthritis Risk Loci." Nature Genetics 42, no 6 (June 2010): 508 14 doi.org/10.1038/ng.582

[27] Schizophrenia Working Group of the Psychiatric Genomics Consortium, "Biological Insights from 108 Schizophrenia-Associated Genetic Loci." Nature 511, no 7510 (July 2014): 421 27 doi.org/10.1038/nature13595

[28] Morris, Andrew P., Benjamin F. Voight, Tanya M. Teslovich, Teresa Ferreira, Ayellet V. Segrè, Valgerdur Steinthorsdottir, Rona J. Strawbridge, et al, "Large-Scale Association Analysis Provides Insights into the Genetic Architecture and Pathophysiology of Type 2 Diabetes." Nature Genetics 44, no 9 (September 2012): 981 90 doi.org/10.1038/ng.2383

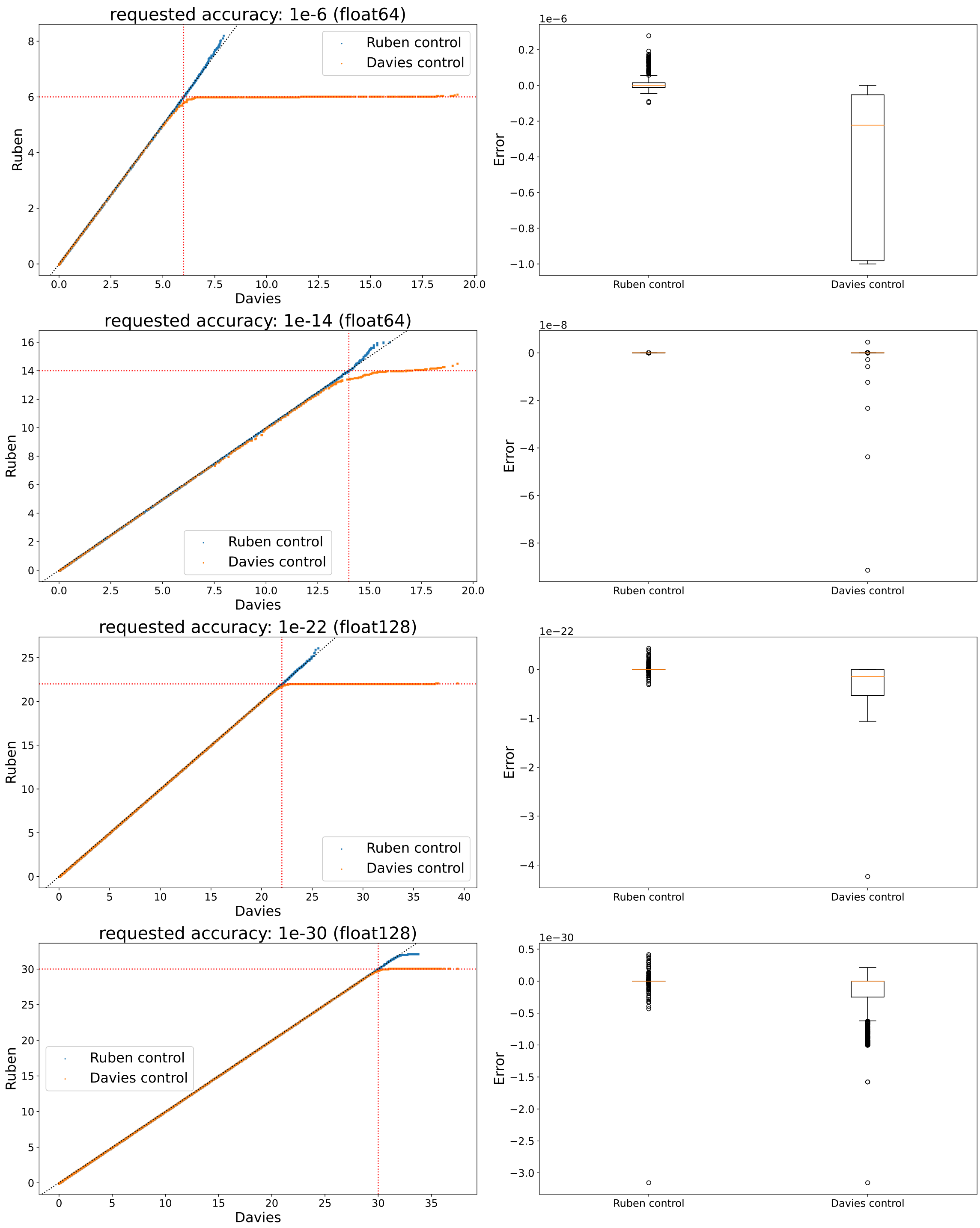

Figure 1: Cross verification of the CDF calculation of a linear combination of χ_1^2 distributions. Combination coefficients are randomly sampled from the range $[0.01, 1]$, evaluation point from $[0.1, 1000]$ and number of coefficients from $[5, 500]$. We took 10000 samples and kept only data points where both algorithms successfully converged error free under 100000 iterations. The left plots show $-log_{10}$ transformed p-values plotted against each other. The right plots absolute errors. Top two rows: Control with *float128*. Bottom two rows: Control with multi-precision arithmetic at 100 digits.

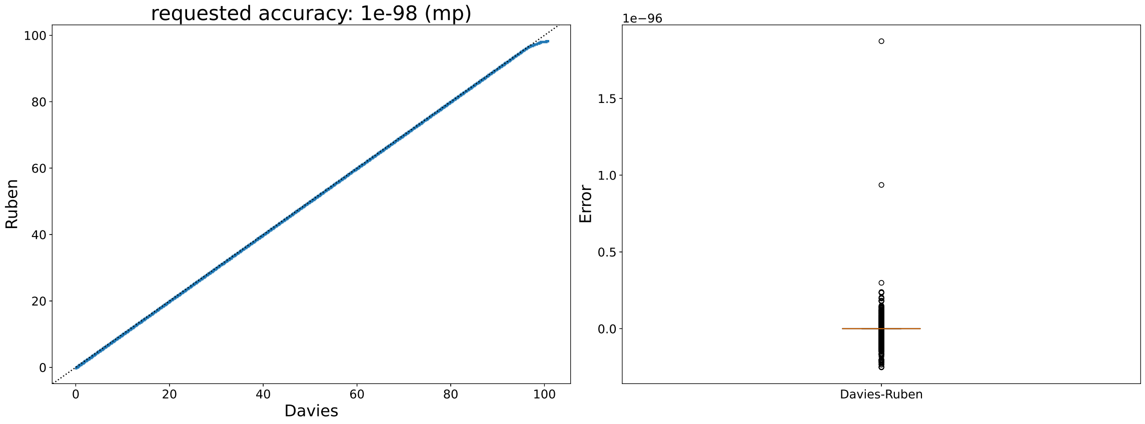

Figure 2: Cross verification of CDF calculation as in figure 1, but both algorithms at 100 digits precision (no control at higher precision available). Left: $-log_{10}$ p-values plotted against each other. Right: Absolute error.

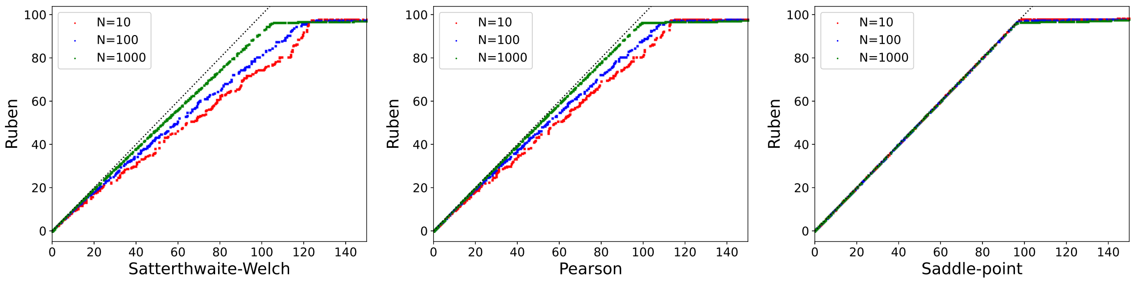

Figure 3: From left to right: Satterthwaite-Welch, Imhof-Pearson's and Saddle-point approximation against Ruben's algorithm for different number of terms in the linear combination of χ_1^2 random variables. Sampling as for figure 1, but with fixed number of coefficients, evaluation point sampled from $[0.1, 2000]$ and 1000 samples in total. Ruben has been run with multi-precision arithmetic at 100 digits. $-\log_{10}$ transformed p-values are plotted against each other.

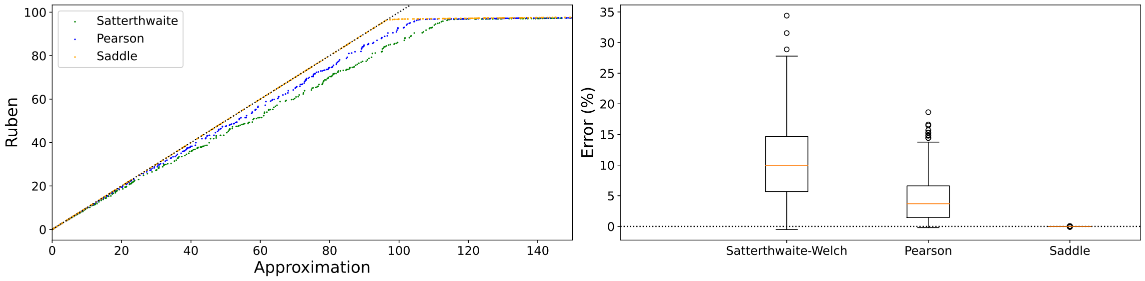

Figure 4: Sampling as for figure 1, but evaluation point from $[0.1, 2000]$. Ruben has been run with multi-precision arithmetic at 100 digits. $-\log_{10}$ transformed p-values are plotted against each other. For the error plot on the right hand side showing the % deviation from Ruben we only considered p-values $> 1e^{-96}$ and < 0.99 under the exact calculation. The maximum observed error of the saddle method is $\approx 1.5\%$.

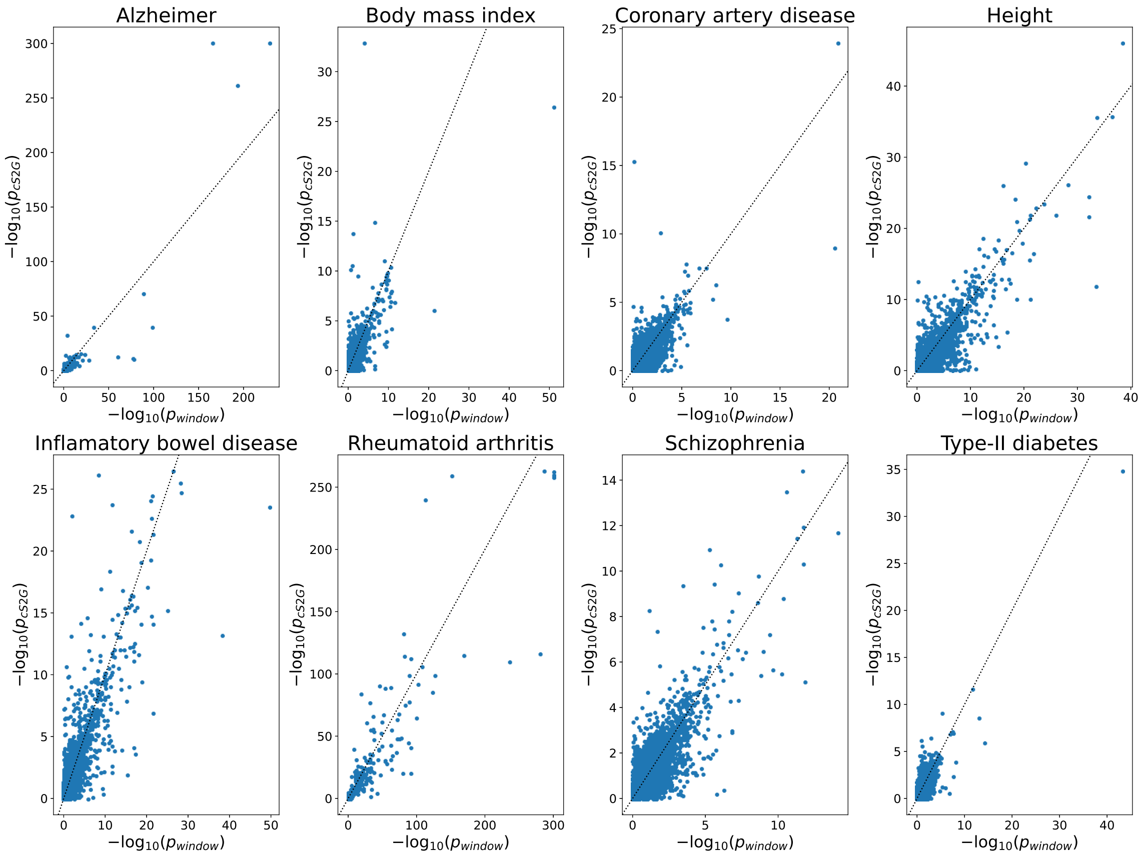

Figure 5: Gene p-values ($-\log_{10}$ transformed) computed for SNPs in gene transcription side plus 50kb window on both sides (x-axis), and via the cS2G SNP to gene linking of [19] (y-axis). In both cases we used the saddle point approximation method to evaluate the cumulative distribution function. For cS2G we incorporated the given linking score by utilizing the weighted statistic introduced in section 2.3.

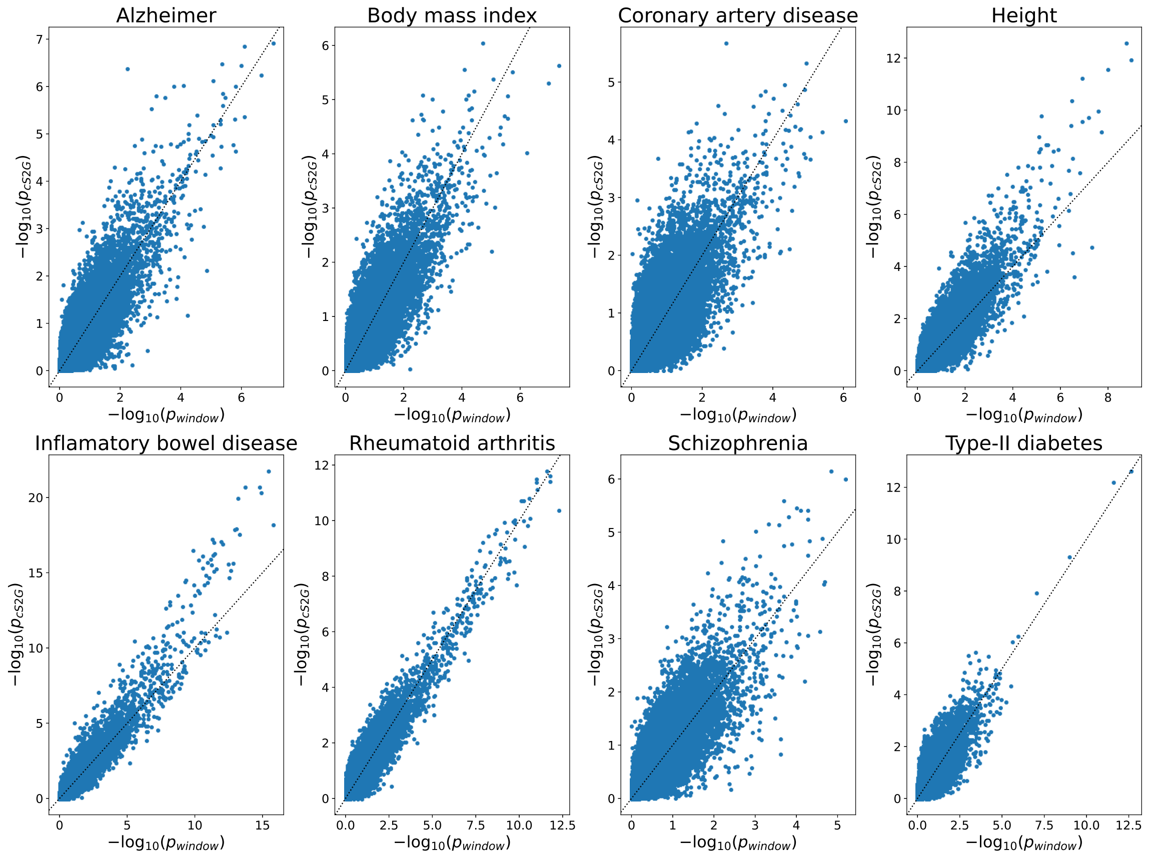

Figure 6: MSigDB pathway p-values ($-\log_{10}$ transformed) computed for SNPs in gene transcription side plus 50kb window on both sides (x-axis), and via the cS2G SNP to gene linking of [19] (y-axis). In both cases we used the saddle point approximation method to evaluate the cumulative distribution function. For the cS2G we incorporated the given linking score by utilizing the weighted statistic introduced in section 2.3.

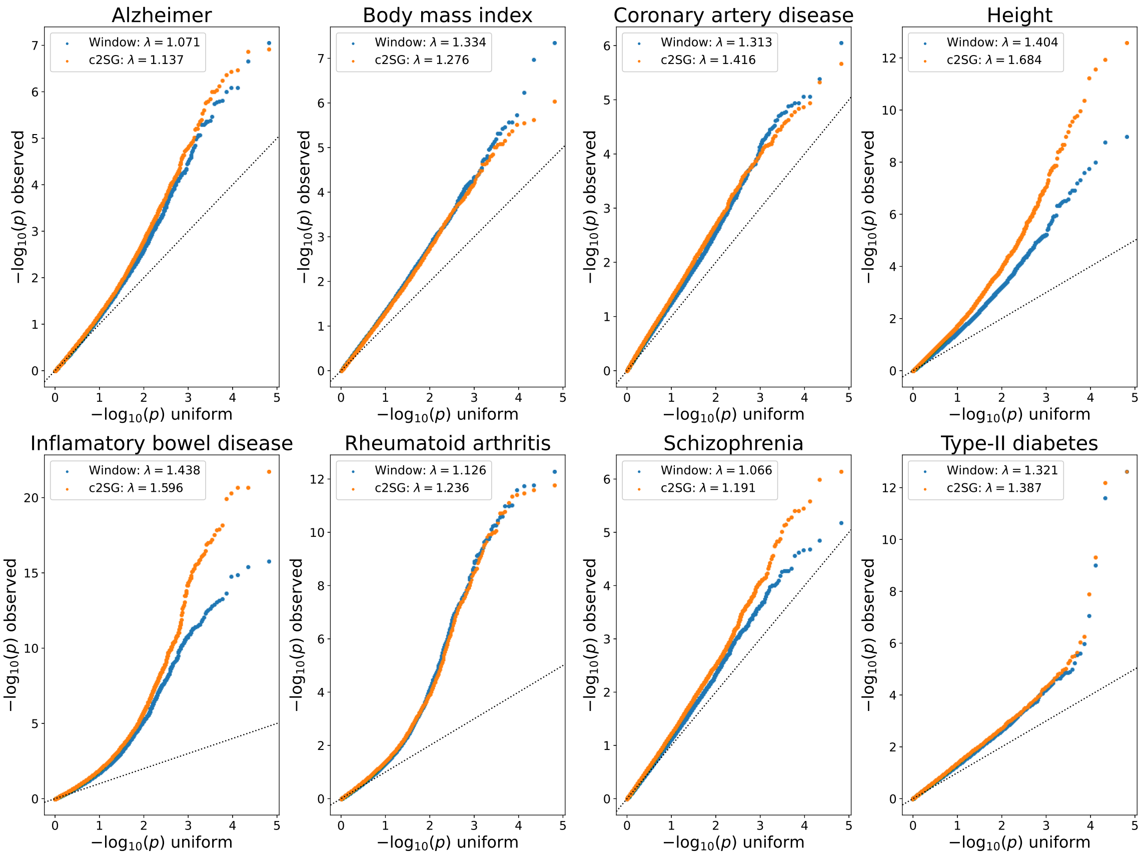

Figure 7: QQ-plots for the MSigDB pathway p-values given in figure 2.3. We observe that for some of the traits biological pathways (Height, Inflamatory bowel disease and Schizophrenia) appear to be significantly more enriched under cS2G.

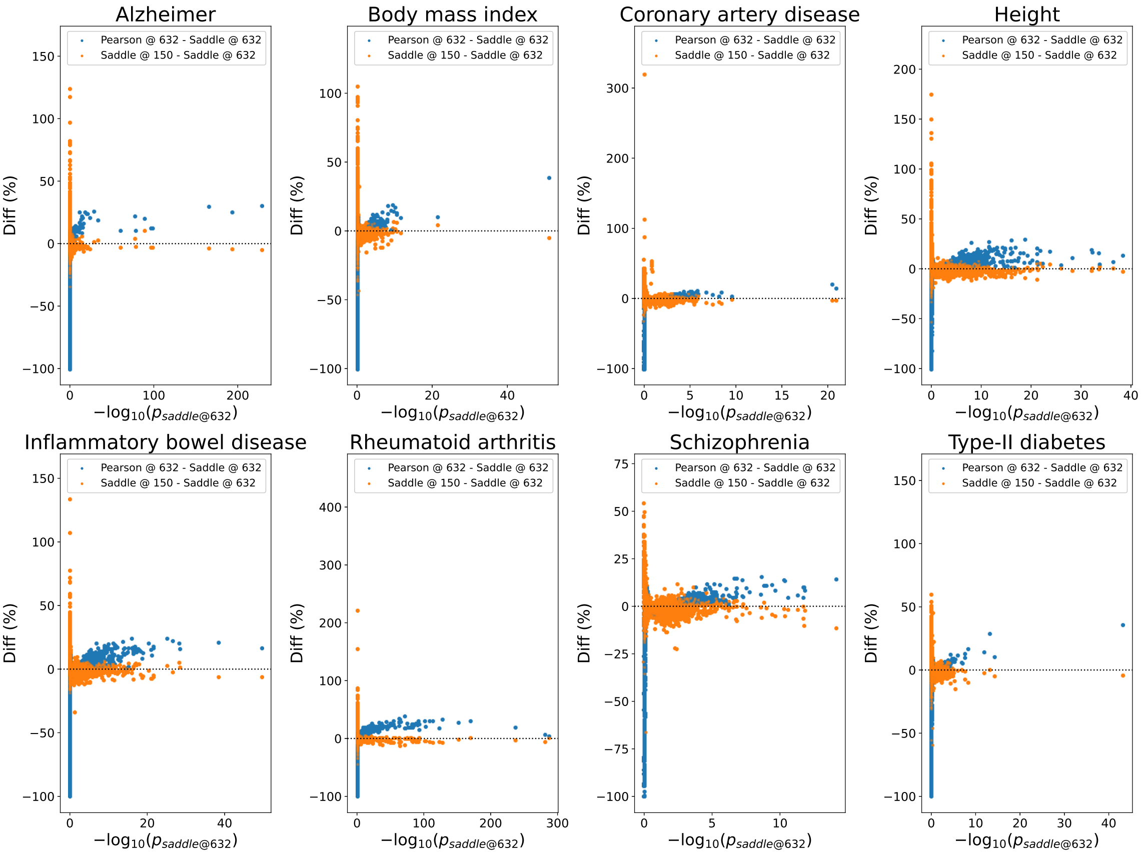

Figure 8: Difference (in %) for gene scores ($-\log_{10}$ transformed p-values) computed with different reference panel size (randomly sub-sampled, 150 samples) and method (Pearson) plotted against base line given by full reference panel (632 samples) and saddle point method.

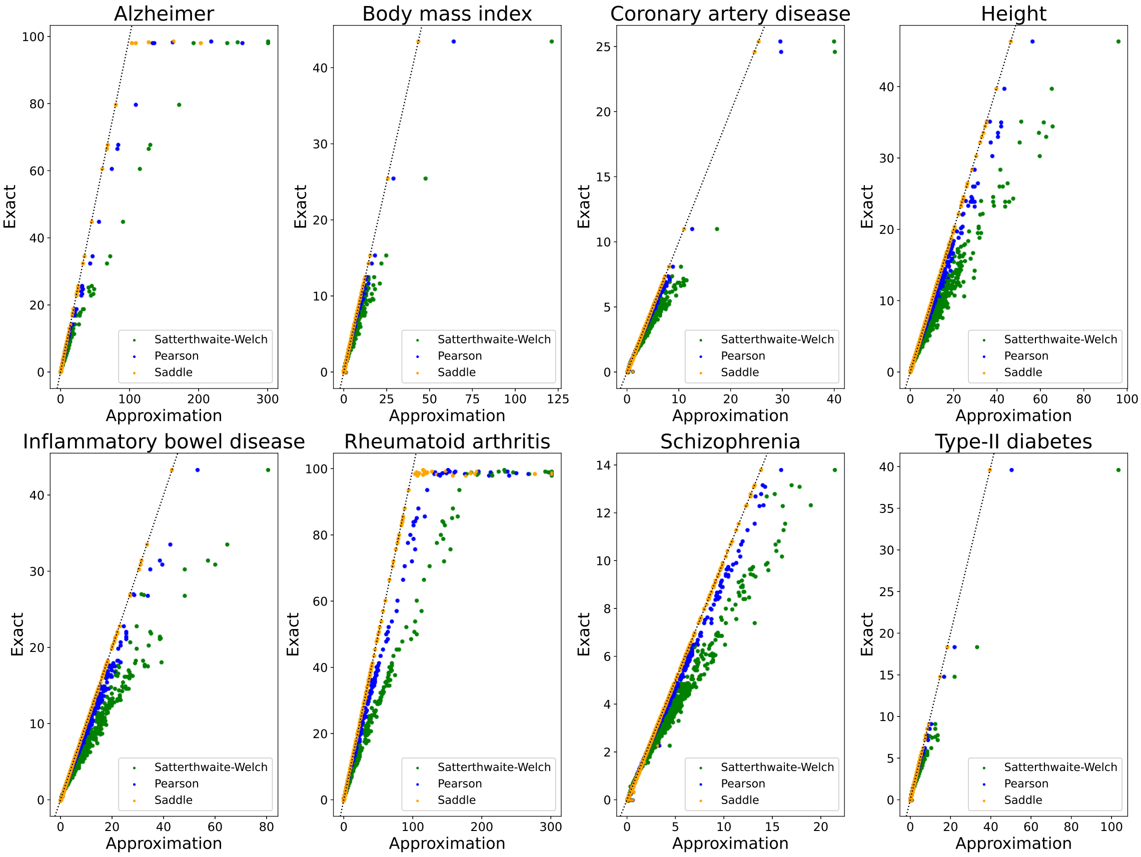

Figure 9: Gene p-values ($-\log_{10}$ transformed) computed via Satterthwaite-Welch (green), Imhof-Pearson (blue) and Saddle-point (orange) approximation plotted against the exact solution for the GWAS listed in table 1. Default settings of *PascalX* have been used.

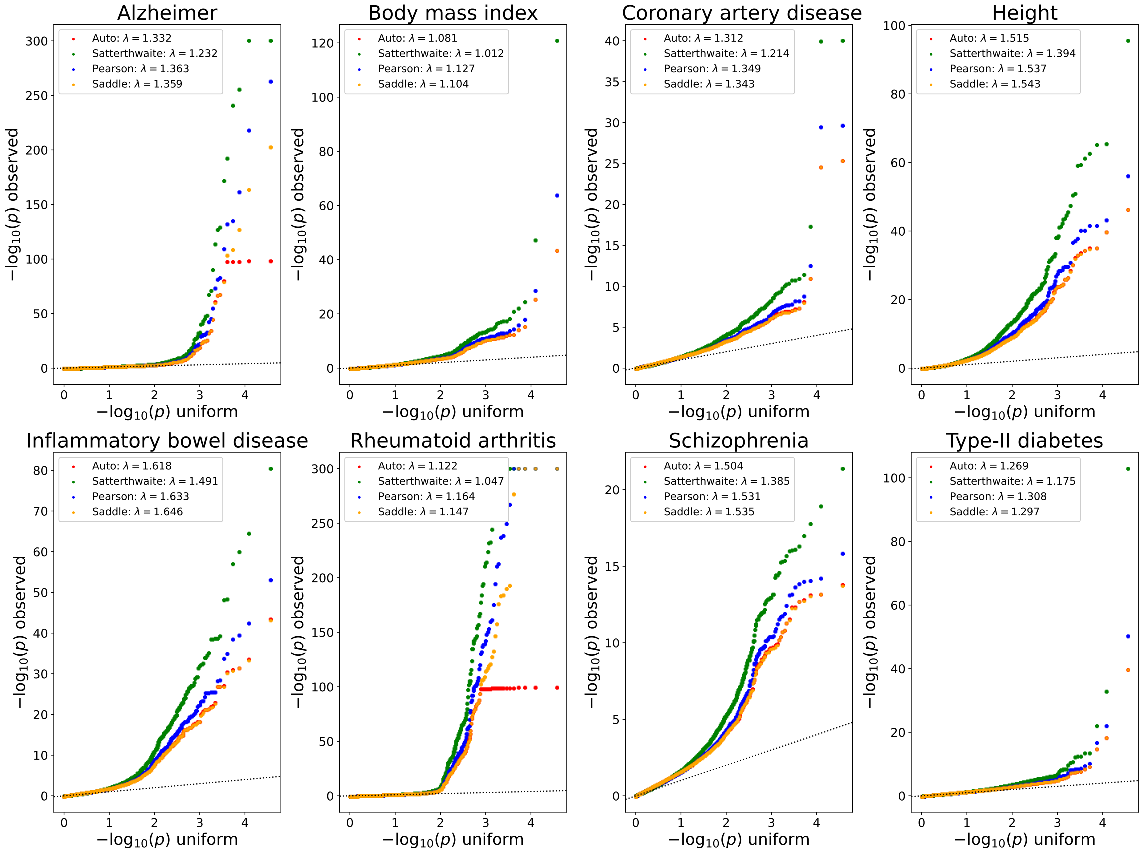

Figure 10: QQ-plots for the PascalX computed gene p-values given in figure 9.

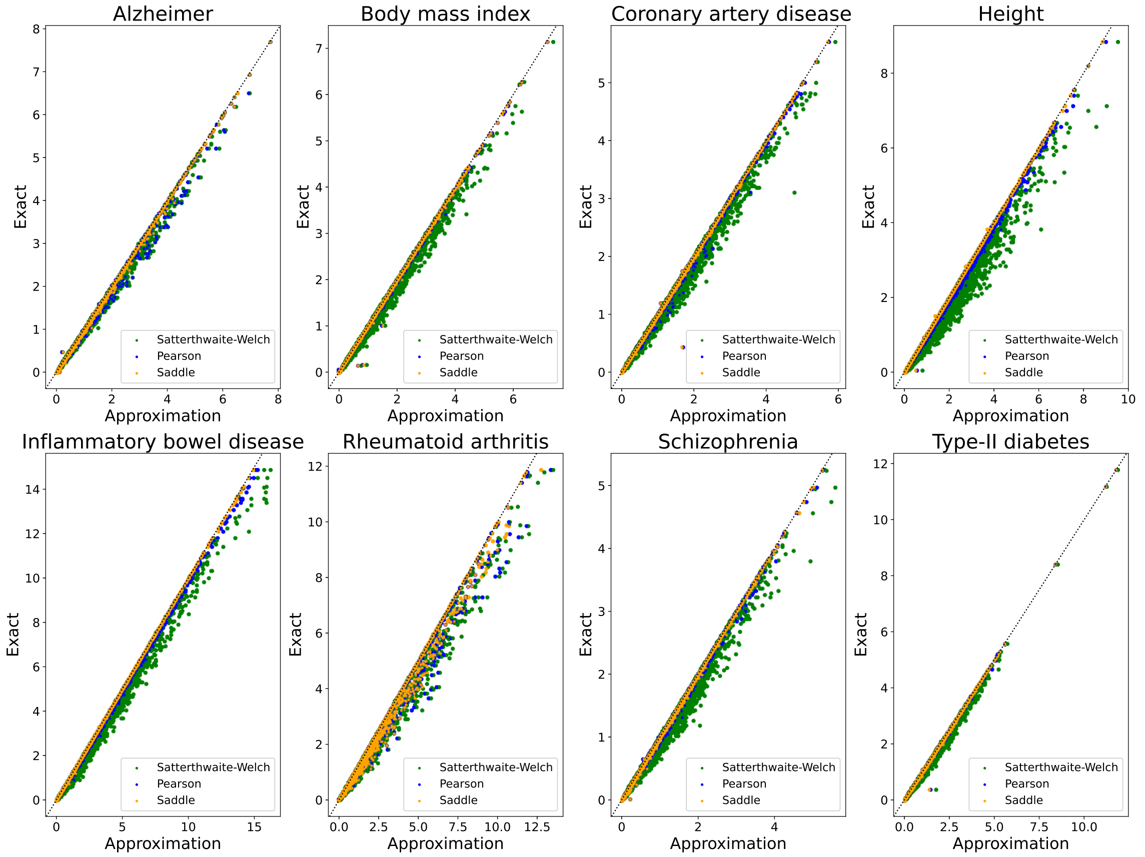

Figure 11: MSigDB pathway p-values ($-\log_{10}$ transformed) computed via Satterthwaite-Welch (green), Imhof-Pearson (blue) and Saddle-point (orange) approximation for the GWAS listed in table 1. The results are plotted against the exact calculation. Default settings of *PascalX* have been used.

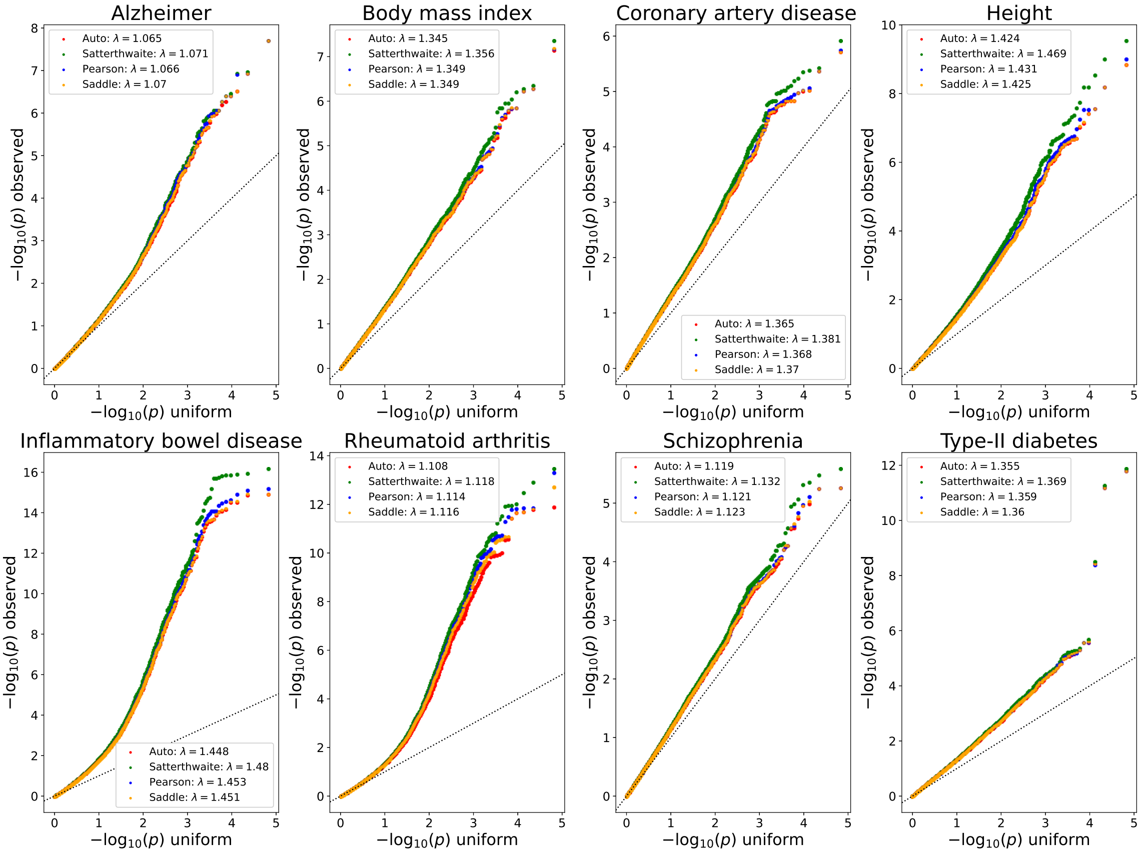

Figure 12: QQ-plots for the PascalX computed pathway p-values given in figure 11.

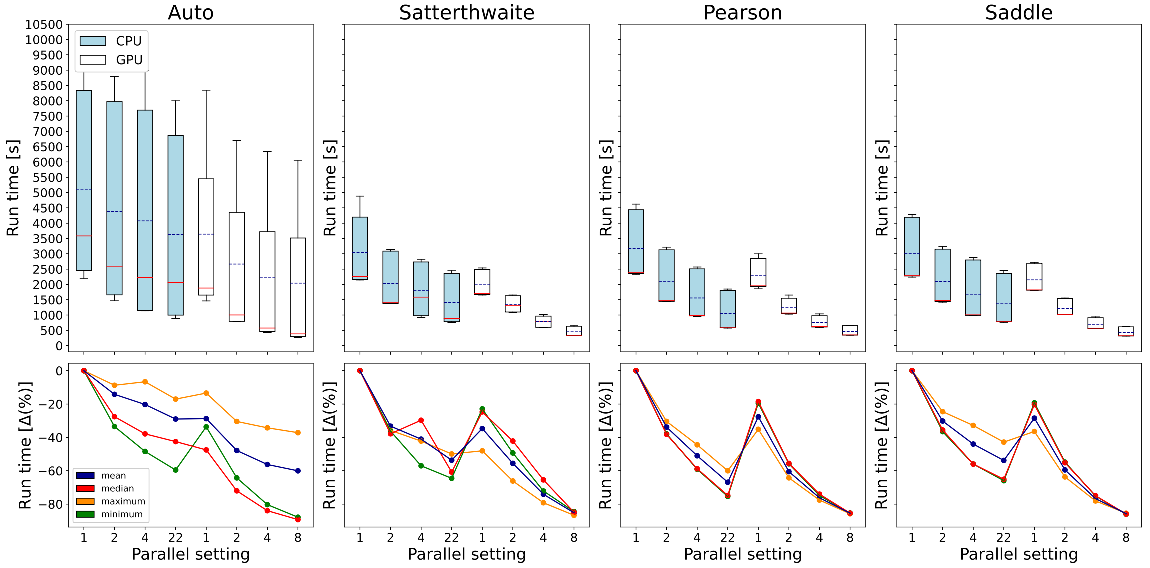

Figure 13: PascalX gene scoring performance for different levels of multi-threading and with or without utilization of a GPU for linear algebra operations (pink box-plots: no GPU; green box-plots: with GPU). The box plots are based on the 8 genome wide association studies listed in table 1. We show the results for the exact calculation (auto), and the approximation based on the methods of Satterthwaite-Welch and Imhof-Pearson, and the Saddle-point method. The mean run-times are indicated with orange markings.

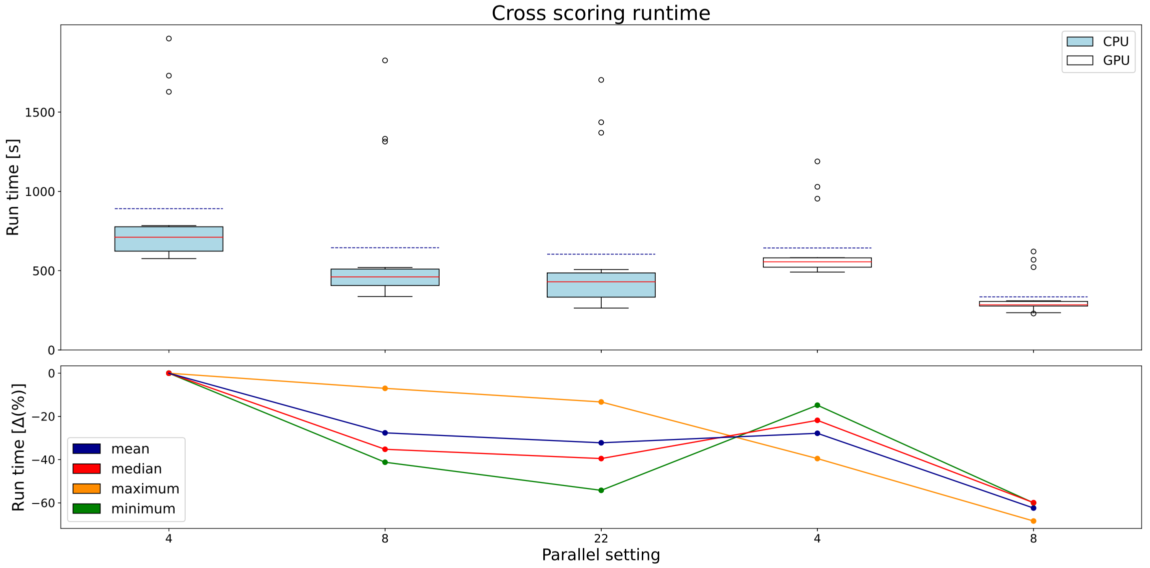

Figure 14: PascalX cross-scoring performance for different levels of multi-threading and with or with-out utilization of a GPU for linear algebra operations (pink box-plots: no GPU; green box-plots: with GPU). The box plots are based on the cross scoring execution run time for each unique pair of 6 genome wide association studies listed in table 1. Mean run-times are marked in orange and outliers with circles.

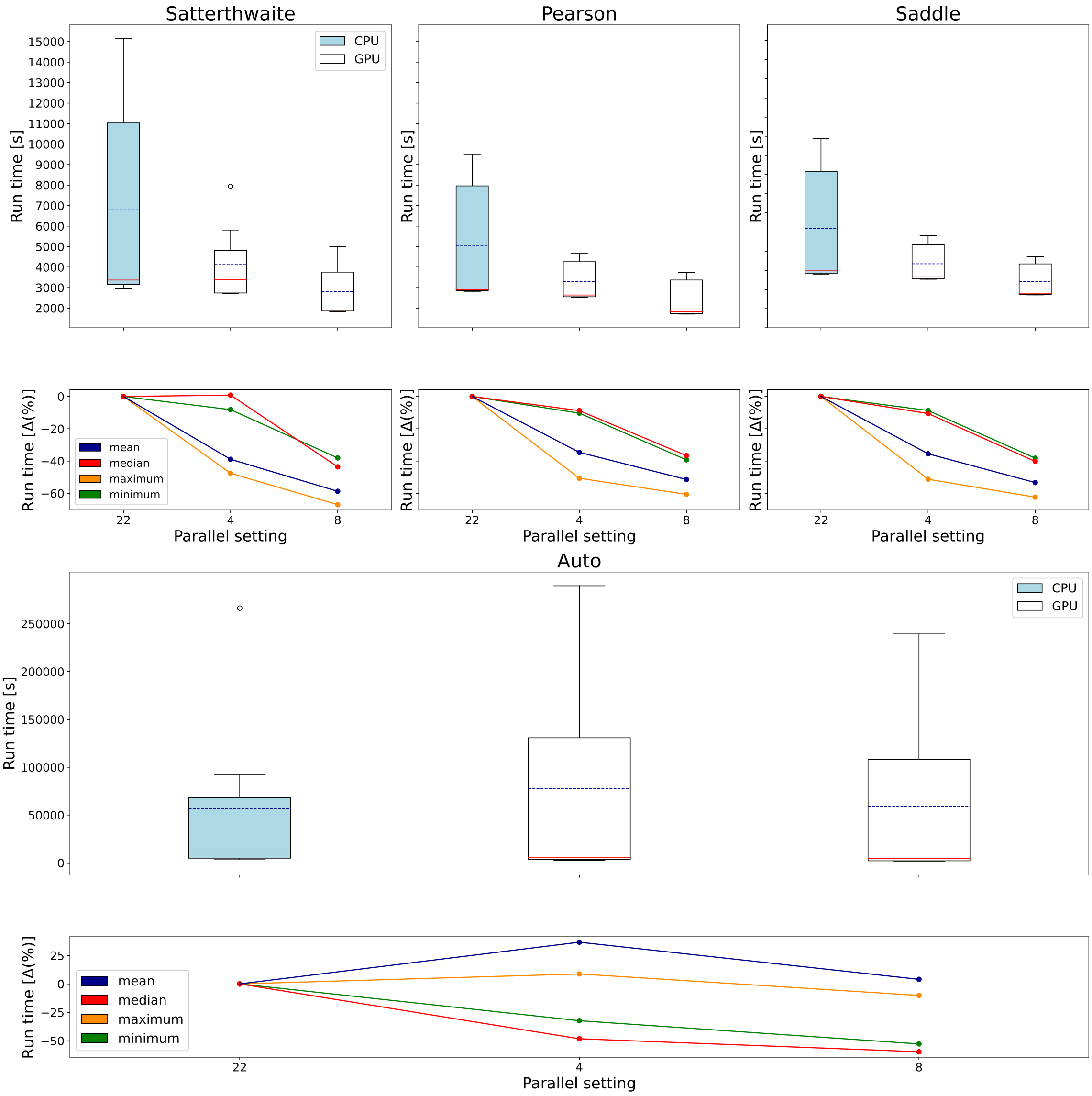

Figure 15: PascalX pathway scoring performance for different levels of multi-threading and with or with-out utilization of a GPU for linear algebra operations (pink box-plots: no GPU; green box-plots: with GPU). The box plots are based on the 8 genome wide association studies listed in table1. We show the results for the exact calculation (top plot), and the approximation based methods of Satterthwaite-Welch and Imhof-Pearson, and the Saddle-point method (bottom plots). All pathways of MSigDB v7.4 have been tested. Mean run-times are marked in orange and circles indicate outliers.

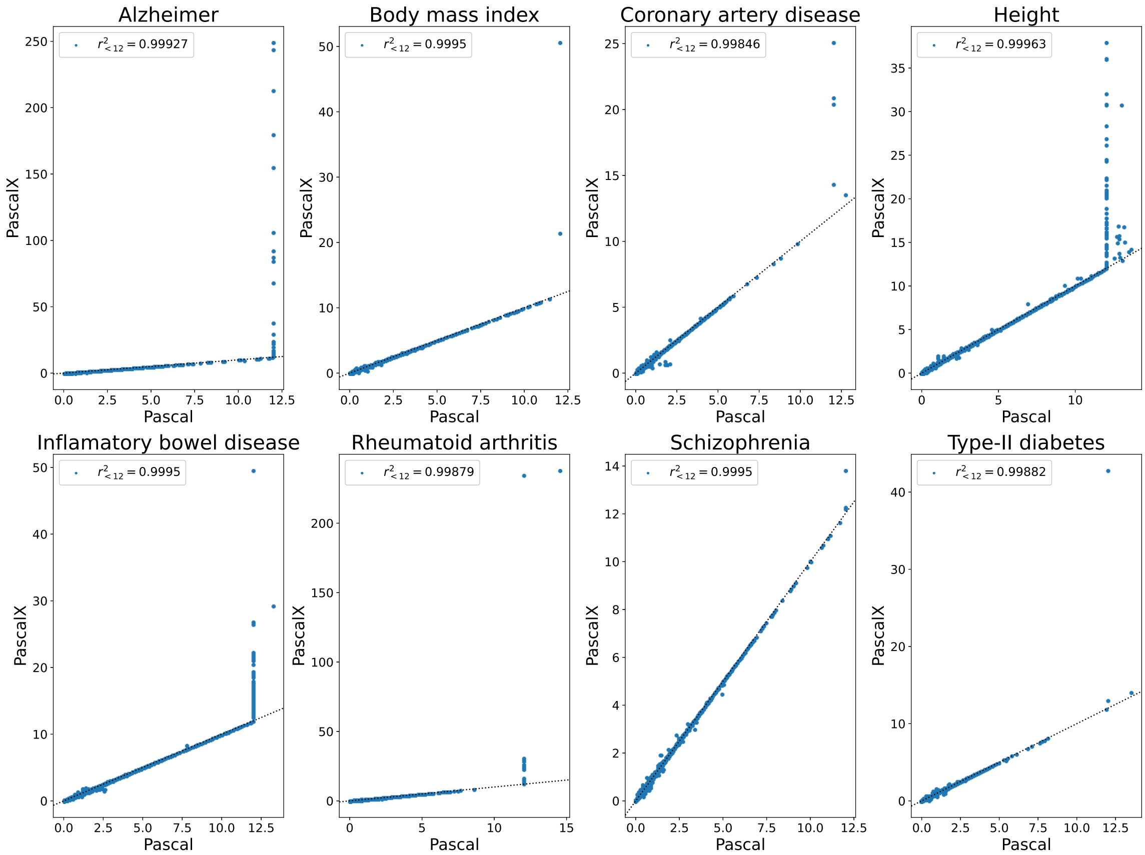

Figure 16: Gene scores ($-\log_{10}$ transformed p-values) for PascalX and the original Pascal implementation plotted against each other. r^2 value calculation includes only datapoints with a PascalX gene score < 12. As is evident, the original weighted χ^2 based Pascal implementation starts to break down around a $-log_{10}$ p-value of 12. PascalX scores have been calculated with a saddle point approximation.

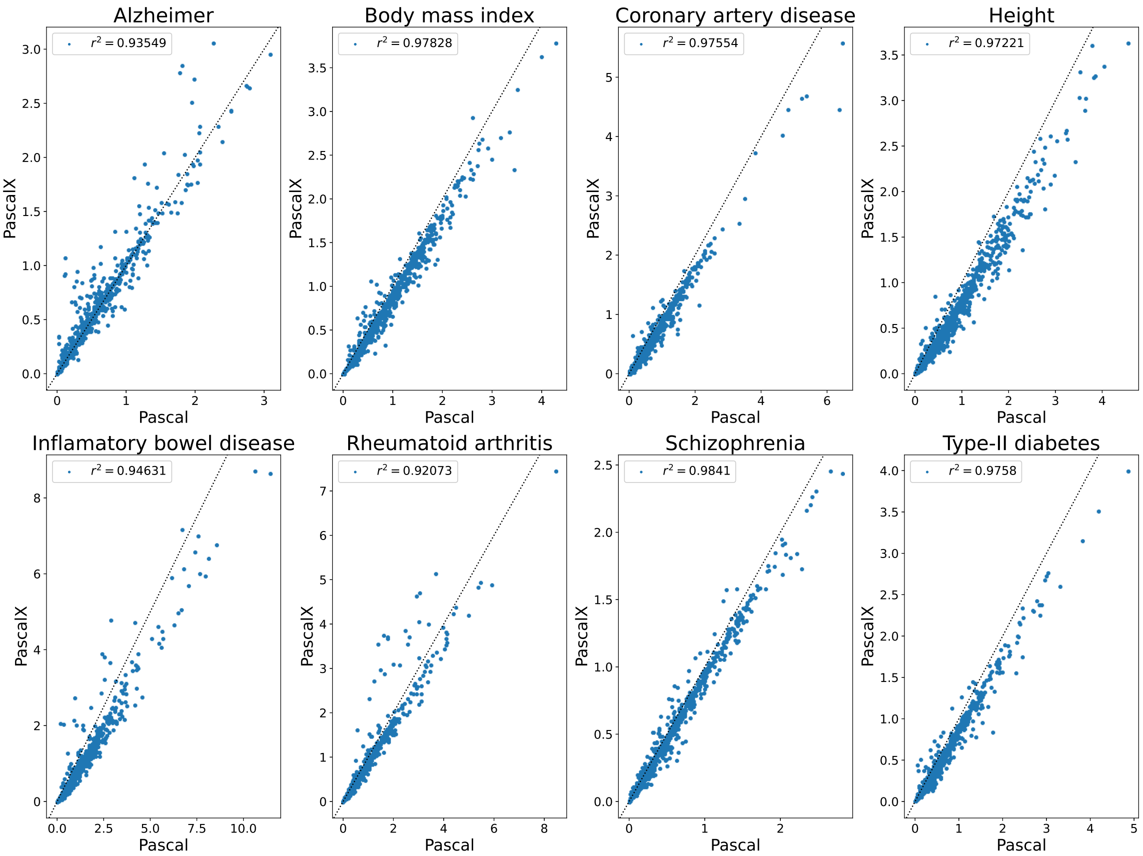

Figure 17: Pathway scores ($-\log_{10}$ transformed p-values) for PascalX and the original Pascal implementation plotted against each other. As in figure 16 we used the saddle point approximation for PascalX. We considered the Biocarta, KEGG and Reactome pathway subset of MSigDB included in the original Pascal package (1077 pathways). Merge distance for gene fusion was set to 1 Mb. We observe that for the highly powered Alzheimer and Rheumatoid arthritis GWAS there is an impact onto the pathway scores despite QQ normalization. That is, being able to resolve the ordering of significant p-values matters.

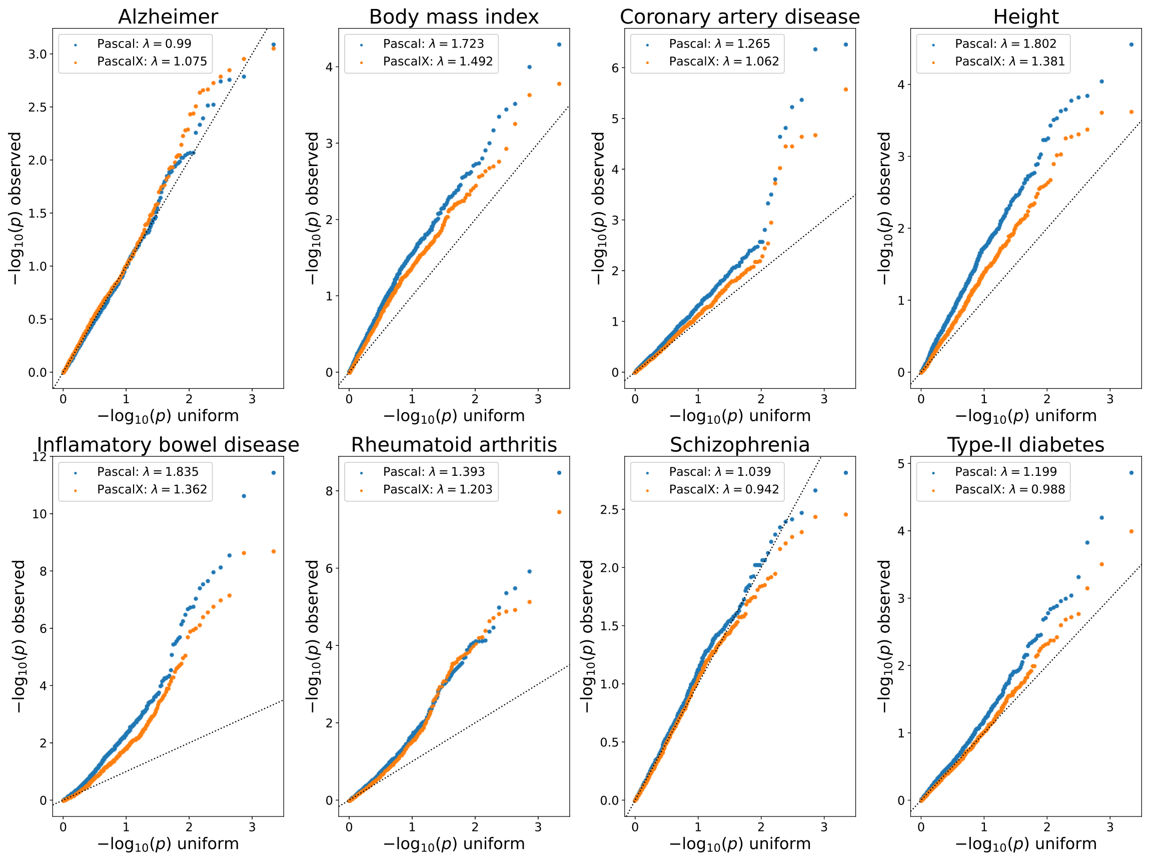

Figure 18: QQ-plot for pathway scores given in figure 17. Note that the new PascalX implementation yields less inflated results.

GWAS	Satterthwaite				Pearson				Saddle			
	**all**	$p < 16$	$16 < p < 50$	$p > 50$	**all**	$p < 16$	$16 < p < 50$	$p > 50$	**all**	$p < 16$	$16 < p < 50$	$p > 50$
ALZ	0.999407	0.999405	0.968421	-	0.999569	0.999568	0.980451	-	0.999856	0.999855	1.0	-
BMI	0.998974	0.998974	-	-	0.998543	0.998543	-	-	0.999779	0.999779	-	-
CAD	0.999289	0.999289	-	-	0.999434	0.999434	-	-	0.999767	0.999767	-	-
H	0.999549	0.999546	0.943199	-	0.999439	0.999435	0.975194	-	0.999977	0.999977	1.0	-
IBD	0.999651	0.999648	0.806175	-	0.999768	0.999767	0.926910	-	0.999935	0.999934	1.0	-
RA	0.999229	0.999215	0.980199	0.898635	0.999266	0.999253	0.991934	0.94282	0.999960	0.999959	1.0	1.0
SCZ	0.999420	0.999420	-	-	0.999556	0.999556	-	-	0.999657	0.999657	-	-
T2D	0.999192	0.999192	-	-	0.999286	0.999286	-	-	0.999771	0.999771	-	-

Table 11: Spearman correlation between gene scores obtained via different approximation scoring methods and the exact calculation. We considered different p-value regimes and took only regimes into account with more than 10 genes present. We also removed genes with p-values smaller than $1e^{-95}$ in order to not reach the precision limitation of the exact calculation. "-" indicates that the GWAS has insufficient data for the regime. Trait codes are defined in the section *"Data availability"*.

GWAS	Satterthwaite			Pearson			Saddle		
	**all**	$p < 4$	$p \geq 4$	**all**	$p < 4$	$p \geq 4$	**all**	$p < 4$	$p \geq 4$
ALZ	0.999554	0.999551	0.947617	0.999920	0.999919	0.963985	0.999939	0.999939	0.998328
BMI	0.999332	0.999329	0.876633	0.999886	0.999886	0.993853	0.999903	0.999903	0.996073
CAD	0.999140	0.999137	0.892986	0.999906	0.999906	0.992559	0.999945	0.999945	0.999562
H	0.997839	0.997811	0.913062	0.999808	0.999806	0.989237	0.999960	0.999959	0.999690
IBD	0.998791	0.998715	0.974895	0.999927	0.999922	0.997607	0.999984	0.999983	0.999918
RA	0.998843	0.998806	0.936947	0.999466	0.999449	0.958019	0.999571	0.999557	0.973316
SCZ	0.999018	0.999017	0.881119	0.999900	0.999899	0.993007	0.999929	0.999929	0.986014
T2D	0.999248	0.999245	0.954118	0.999927	0.999926	0.992398	0.999944	0.999944	0.998914

Table 12: Spearman correlation between pathway scores obtained from the approximate gene scores and exactly calculated gene scores.
